# QKI‐6 inhibits bladder cancer malignant behaviours through down‐regulating E2F3 and NF‐κB signalling

**DOI:** 10.1111/jcmm.14481

**Published:** 2019-08-26

**Authors:** Fei Shi, Zheng Deng, Zheng Zhou, Chen‐Yi Jiang, Rui‐Zhe Zhao, Feng Sun, Di Cui, Xiao‐Yu Bei, Bo‐Yu Yang, Qian Sun, Xing‐Jie Wang, Qi Wu, Shu‐Jie Xia, Bang‐Min Han

**Affiliations:** ^1^ Department of Urology, School of Medicine Shanghai General Hospital, Shanghai Jiao Tong University Shanghai China; ^2^ Department of Urology Shanghai General Hospital Affiliated to Nanjing Medical University Shanghai China; ^3^ Institute of Urology Shanghai Jiao Tong University Shanghai China

**Keywords:** bladder cancer, E2F3, gene regulation, QKI, survival, tumour progression

## Abstract

Quaking homolog (QKI) is a member of the RNA‐binding signal transduction and activator of proteins family. Previous studies showed that QKI possesses the tumour suppressor activity in human cancers by interacting with the 3'‐untraslated region (3'‐UTR) of various gene transcripts via the STAR domain. This study first assessed the association of QKI‐6 expression with clinicopathological and survival data from bladder cancer patients and then investigated the underlying molecular mechanisms. Bladder cancer tissues (n = 223) were subjected to immunohistochemistry, and tumour cell lines and nude mice were used for different in vitro and in vivo assays following QKI‐6 overexpression or knockdown. QKI‐6 down‐regulation was associated with advanced tumour TNM stages and poor patient overall survival. QKI‐6 overexpression inhibited bladder cancer cell growth and invasion capacity, but induced tumour cell apoptosis and cell cycle arrest. Furthermore, ectopic expression of QKI‐6 reduced tumour xenograft growth and expression of proliferation markers, Ki67 and PCNA. However, knockdown of QKI‐6 expression had opposite effects in vitro and in vivo. QKI‐6 inhibited expression of E2 transcription factor 3 (E2F3) by directly binding to the E2F3 3'‐UTR, whereas E2F3 induced *QKI‐6* transcription by binding to the *QKI‐6* promoter in negative feedback mechanism. QKI‐6 expression also suppressed activity and expression of nuclear factor‐κB (NF‐κB) signalling proteins in vitro*,* implying a novel multilevel regulatory network downstream of QKI‐6. In conclusion, QKI‐6 down‐regulation contributes to bladder cancer development and progression.

## 1 INTRODUCTION

Bladder cancer remains a significant health problem in both men and women worldwide.^1^ In China alone, the incidence of bladder cancer has increased in the past decade.^2^ Approximately 70% of tumour lesions are non‐muscle‐invasive bladder cancer; however, these tumours can undergo recurrence and become invasive.^3^ Thus, any tumour lesions in the urinary bladder should undergo long‐term surveillance. Treatment of bladder cancer depends on the tumour stage. For example superficial tumour lesions can be surgically removed by a resectoscope^4^ followed by immunotherapy with Bacillus Calmette‐Guérin.^5^ Bladder cancer with deep muscle invasion could be treated with cystectomy or radical cystectomy followed by radiation and chemotherapy, whereas micro‐metastasized disease should be treated with neoadjuvant chemotherapy.^4^ Risk factors for developing bladder cancer include tobacco smoking and occupational exposure to carcinogens (like benzidine).^6^ These risk factors can alter gene expression and promote bladder carcinogenesis.^6^ To date, despite improvements in surgical techniques and chemotherapeutic agents, more than 50% of bladder cancer patients still develop metastatic disease and suffer death.^7^ Thus, there is an urgent need to identify biomarkers for early detection, prognosis and treatment responses and increase our understanding of the molecular mechanisms underlying bladder cancer pathogenesis.

Quaking homologue (QKI) belongs to the RNA‐binding signal transduction and activator of RNA (STAR) protein family.^8^ All QKI protein isoforms contain an identical GSG/STAR domain and possess identical RNA‐binding specificities.^9^ QKI‐5 is the major nuclear isoform expressed during embryogenesis and its expression declines after birth, whereas QKI‐6 and QKI‐7 isoforms are expressed during late stages of embryogenesis with peak expression occurring during myelination.^10^ However, these isoforms have a distinct C terminal as a result of alternative splicing of premature mRNA.^11^ QKI proteins can selectively interact with a short DNA sequence in their target gene promoters, termed the QKI response element (QRE; 5'‐ACUAAY…N_1‐20_ UAAY‐3') of their targeting genes (ie QKI interactions with RNAs in the 3'‐UTR via the STAR domain). However, the structure of the entire STAR domain with bound RNA remains unknown, especially in cancer cells.^12^ A previous study showed that QKI directly regulates the expression of p27 and cyclinD1 to form a negative feedback loop with E2 transcription factor 1 (E2F1), indicating that QKI may be involved in cell cycle regulation and cell differentiation.^13^ More recent studies demonstrated that QKI possesses tumour suppressor activity in various human cancers, including glioblastoma, and prostate, colon, lung, gastric, oral and kidney cancers.^9^, ^11^, ^14^, ^15^, ^16^, ^17^, ^18^

E2 transcription factor 3 (E2F3) is localized at chromosome 6p22, where gene amplification occurs in approximately 9% of bladder cancers and is associated with a higher bladder tumour stage and grade, suggesting that E2F3 is an oncogene.^19^ Moreover, phosphorylation of the Rb protein permits uncomplexed E2F3 to promote cell cycle progression from the G1 to S phase in bladder cancer.^20^ A previous study demonstrated that post‐transcriptional regulation of E2F3 by miR‐125b could suppress bladder cancer progression.^21^ In addition, the NF‐κB pathway involves phosphorylation, ubiquitination and degradation of IκBα and subsequent nuclear translocation of the p50 and p65 subunits of NF‐κB, which facilitates target gene transcription. Thus, activation of NF‐κB signalling is associated with cancer development, metastasis and chemical resistance, and abnormal constitutive activation of NF‐κB signalling has been demonstrated in human cancers, including lung, prostate, breast and bladder cancers. Mutations of various oncogenes in combination with an inflammatory microenvironment can lead to aberrant NF‐κB activation.^22^, ^23^

In this study, we assayed pan‐QKI expression in bladder cancer compared to normal tissue samples and determined the association of QKI‐6 expression with clinicopathological data and patient survival. We then investigated the effects of QKI‐6 overexpression or knockdown on bladder cancer cell malignant behaviours in vitro and in nude mouse xenografts. Our findings provide novel insight into the role of QKI‐6 in bladder cancer development and progression and postulate that targeting the QKI‐6‐E2F3 interaction and NF‐κB signalling pathway could serve as a therapeutic strategy to clinically control bladder cancer.

## 2 MATERIALS AND METHODS

### 2.1 Patients and tissue samples

This study was approved by the ethics committee of Shanghai General Hospital (Shanghai, China). In brief, we retrospectively collected tissue samples and the corresponding clinicopathological data (Table S1) from 223 patients with histologically diagnosed bladder cancer from our hospital (Shanghai, China). Written informed consent was obtained from all patients included in the study. These patients were diagnosed with bladder cancer according to cystoscopy criteria and staged with muscle‐invasive classification.^6^ Both tumour and matched adjacent normal tissues were collected and histologically confirmed by the Department of Pathology, Shanghai General Hospital. Paraffin blocks from each patient were retrieved from the Pathology Department and subjected to tissue microarray. These patients were followed for up to five years.

### 2.2 Immunohistochemistry

Tissue microarray sections (4 μmol/L) were prepared for immunohistochemistry. Specifically, sections were deparaffinized in xylene and rehydrated in a series of ethanol solutions (100%‐50%) and in tap water. Endogenous peroxidase activity was blocked in 0.75% H_2_O_2_ in phosphate‐buffered saline (PBS) for 50 minutes. The sections were then incubated in 5% bovine serum albumin (BSA) in PBS for 30 minutes at room temperature and incubated with a mouse anti‐human QKI antibody (1:300; Sigma Chemicals, St. Louis, MO) or a mouse anti‐human E2F3 antibody (1:500; Abcam, London, UK) at 4°C overnight. Next, immune detection followed a three‐step protocol with a streptavidin‐horseradish peroxidase complex and visualization by 3, 3‐diaminobenzidine, according to a previous study.^14^ The immunostained tissue microarray sections were reviewed and scored using a cut‐off value of 30% as low versus high QKI‐6 expression in these tissue specimens.

### 2.3 Cell lines and culture

Bladder cancer 5637, T24, 253J, RT4, TCCSUP and J82 cell lines, a normal bladder epithelial cell line SV‐HUC‐1, and a normal kidney cell line HEK‐293T were cultured in Roswell Park Memorial Institute medium‐1640 supplemented with 10% foetal bovine serum (FBS) and 0.1% penicillin‐streptomycin in 25 cm^2^ plastic cell culture flasks (all from Invitrogen, Carlsbad, CA) in a humidified incubator with 5% CO_2_ at 37°C. Various concentrations (3‐1000 μmol/L) of pyrrolidinedithiocarbamate (PDTC; Targetmol, Boston, MA) or its vehicle (culture medium plus the solvent) were added to the cells for 6 hours prior to additional experimentation.^24^

### 2.4 Plasmid carrying QKI‐6 cDNA or shRNA and lentiviral transfection

Plasmids carrying either QKI‐6 shRNA or negative control shRNA were constructed and lentivirus was generated in HEK‐293 cells using OBiO (Shanghai, China). The plasmid containing QKI‐6 cDNA was also obtained from OBiO. To knockdown or overexpress QKI‐6, bladder cancer cells were seeded at a density of 1 × 10^5^ in 6‐well plates and grown to approximately 75% confluency. Next, the culture medium was removed and fresh culture medium consisting of either lentiviral particles containing QKI‐6 shRNA, negative control shRNA, QKI‐6 cDNA or negative control was added using FuGENE 6 (Roche, Indianapolis, IN) according to the manufacturer's instructions. Cells were then further cultured in an incubator at 37°C with 5% CO_2_. After 24 hours transduction, the culture medium containing viral particles was removed and replaced with fresh medium containing an appropriate concentration of puromycin to promote growth to a sufficient cell number. The cell clones stably expressing QKI‐6 shRNA and CMV‐QKI‐6 were selected and expanded. Western blot and quantitative real‐time polymerase chain reaction (qRT‐PCR) analyses were used to evaluate infection efficiency. The positive clones were selected for in vivo experiments after 7 days.

### 2.5 Protein extraction and Western blot analysis

Total cellular protein was extracted from bladder cancer cells using radioimmunoprecipitation assay buffer (Cell Signaling Technology, Danvers, MA). Protein concentrations in these samples were assayed using the bicinchoninic acid protein assay kit (NCM, Suzhou, China), and 60 μg of protein from each sample was resolved by 10% sodium dodecyl sulphate‐polyacrylamide gel electrophoresis (SDS‐PAGE) and transferred onto polyvinylidene fluoride (PVDF) membranes (Millipore, Billerica, MA). For Western blot analysis, membranes were blocked in 5% skimmed milk or BSA‐PBS solution for 1 hour at room temperature and then incubated at 4°C overnight with a primary antibody. Primary antibodies included: anti‐p21, p27, cyclin D1, cyclin E1, cyclin B, caspase‐3, poly (ADP ribose) polymerase (PARP), nuclear factor‐κB (NF‐κB) pathway antibody sample kit, β‐actin (Cell Signaling Technology), β‐tubulin (Cell Signaling Technology), anti‐cyclin A1 + A2, E2F3 (Abcam, Cambridge, MA), QKI (Sigma Chemicals), QKI‐6 (1:750, Millipore Corporation, CA), caspase‐9, caspase‐12 and Ki‐67 (Novus Biologicals, Littleton, CO). The membranes were then subsequently incubated with horseradish‐peroxidase (HRP) conjugated secondary antibodies (Santa Cruz Biotechnology, Santa Cruz, CA) for 2 hours at room temperature and protein bands were visualized using the enhanced chemiluminescence reagent (Thermo Scientific, Waltham, MA).

### 2.6 qRT‐PCR

Total cellular RNA was isolated from cultured cells using Trizol reagent (Invitrogen) and reverse transcribed into cDNA using the M‐MLV assay kit (Invitrogen) according to the manufacturer's instructions. These cDNA samples were subjected to qPCR amplification of different genes with their primer sequences (Table S1) in 10 µL of the qPCR mixture in a Bio‐Rad PCR amplifier (Hercules, CA). The qPCR mixture included 5 μL SYBR Premix Ex Taq (Vazyme, Nanjing, China), 0.5 μL of each primer (10 pmol/μL), 1 μL cDNA template and 3 μL ddH_2_O. Amplification conditions were initially set at 95°C for 5 minutes and then 40 cycles of 95°C for 5 seconds and 65°C for 30 seconds. The results were analysed using the Applied Biosystems QuantStudio 7 Flex software using 2^−(Ct‐Cc)^ (Ct and Cc were the mean threshold cycle differences after normalizing to β‐actin).

### 2.7 Cell viability CCK‐8 assay

To assess cell proliferation, we first seeded bladder cancer cells after gene transfection at a density of 2 × 10^3^ cells/well into 96‐well plates and cultured the cells for 12, 24 or 48 hours. At the end of each experiment, 20 μL/well of the CCK‐8 reagent (NCM) was added to each well and cells were further cultured for 2 hours. Absorption values were measured at 490 nm using the multi‐well plate reader (BioTek, Winooski, VT). Cell growth curves were plotted according to the average absorption values of each experiment. Experiments were conducted in triplicate and repeated at least three times.

### 2.8 Tumour cell colony formation assay

Following QKI‐6 cDNA or shRNA infection, cells were seeded in 6‐well plates at 500 cells/well and grown for 14 days. The cell growth medium was refreshed every three days, and at the end of the experiments cells were stained with 0.1% crystal violet for 10 minutes at room temperature. Cell colonies with more than 50 cells were counted under an inverted microscope (Leica, Wetzlar, Germany). The experiment was conducted in triplicate and repeated at least once.

### 2.9 Tumour cell transwell invasion assay

Following QKI‐6 cDNA or shRNA infection, cells were seeded into the upper chambers of the Transwell (Corning, Corning, NY). The filter was precoated with Matrigel (BD Biosciences, San Jose, CA) in the growth medium without serum, whereas the bottom chamber was filled with growth medium containing 20% foetal calf serum. The cells were grown for 24 hours. At the end of experiment, cells remaining on the top of the filter were removed using a cotton swab, and the tumour cells that invaded the low side of the filter were fixed with 10% formalin and stained with 0.1% crystal violet for 10 minutes at room temperature. The numbers of invaded cells were counted under an inverted microscope (Leica, Wetzlar, Germany). The experiment was conducted in triplicate and repeated at least once.

### 2.10 Flow cytometry cell cycle assay

To determine changes in cell cycle distribution, transfected cells were grown, washed twice with PBS, and then fixed for at least 2 hours in 300 μL of PBS and 700 μL of 100% ethanol. Next, cells were centrifuged, re‐suspended in 200 μL of extraction buffer (0.1% Triton X‐100, 45 mmol/L Na_2_HPO_4_, and 2.5 mmol/L sodium citrate), incubated at 37°C for 20 minutes, and then re‐suspended and incubated in PBS containing 40 mg/mL propidium iodide, 0.1 mg/mL RNase (Sigma Chemicals) and 0.1% Triton X‐100 at 37°C for 30 minutes in the dark. Cell cycle distributions were analysed using the Fluorescence‐activated cell sorting Accuri C6 flow cytometer (BD Biosciences, Franklin Lakes, NJ).

### 2.11 Animal experiments

All animal experiments were conducted in accordance with the US Public Health Service Policy on Humane Care and Use of Laboratory Animals and were approved by the Scientific Investigation Board of Shanghai General Hospital. In brief, male nude mice (4‐6 weeks old) were purchased from the Experimental Animal Center of Shanghai General Hospital. Tumour cells were grown and suspended in sterile PBS and then injected subcutaneously into each nude mouse (1 × 10^6^ cells/mouse). The size and incidence of subcutaneous tumour xenografts were recorded every 7 days using the following formula: *V* (mm^3^) = width^2^ (mm^2^) × length (mm)/2. After 42 days, the mice were killed by CO_2_ and cervical dislocation to evaluate tumour incidence, weight and size, as well as immunostaining at the indicated time‐points.

### 2.12 Immunofluorescence

Bladder cancer T24 and 5637 cells were grown on coverslips overnight, washed with PBS, and then fixed in 4% formaldehyde solution for 20 minutes. For immunostaining, cells were permeabilized in 0.1% Triton X‐100 for 15 minutes and then blocked in 5% normal goat serum (1:5) in PBS for 1 hour. Next, cells were incubated with a rabbit anti‐QKI antibody (Sigma, Chemicals) at a dilution of 1:500 or antibodies for other regulatory proteins at room temperature for 30 minutes. Cells were washed with PBS three times and further incubated with DyLight 594 and 488‐conjugated goat antirabbit or antimouse IgG (Thermo Scientific) at a dilution of 1:1000 at room temperature for 30 minutes and then counterstained with 4',6‐diamidino‐2‐phenylindole (DAPI; Sigma Chemicals). Staining was scored under an Olympus IX71 fluorescence microscope (Olympus, Tokyo, Japan) and cell images were captured using the microscope‐equipped CellSens imaging software.

### 2.13 Chromatin immunoprecipitation assay

Formaldehyde cross‐linked proliferating 5637 and T24 cells were immunoprecipitated with control IgG or an anti‐E2F3 antibody (Abcam) and the cell nuclei were lysed in a lysis buffer [50 mmol/L Tris‐HCl (pH 8.0), 10 mmol/L ethylene diaminetetra acetic acid (EDTA), 1% sodium dodecyl sulphate (SDS), 1 mmol/L phenylmethylsulfonyl fluoride, protease inhibitors] and sonicated on ice to obtain 250 to 800 bp DNA fragments. The immunocomplex in the cell nuclei was precleared with the Chip Buffer for 1 hour at room temperature and then incubated with anti‐E2F3 antibody at 4°C overnight. On the next day, the immunocomplex was washed three times with Buffer I [20 mmol/L Tris‐HCl (pH 8.0), 2 mmol/L EDTA, 2 mmol/L EGTA, 150 mmol/L NaCl, 1% NP‐40, 0.5% DOC, 0.2% SDS], Buffer II [20 mmol/L Tris‐HCl (pH 9.0), 2 mmol/L EDTA, 2 mmol/L EGTA, 500 mmol/L NaCl, 1% NP‐40, 0.5% DOC, 0.1% SDS] and Buffer III [50 mmol/L Tris‐HCl (pH 7.5), 2 mmol/L EDTA, 1 mmol/L EGTA, 0.5 mol/L LiCl, 1% NP‐40, 0.7% DOC] and then washed once with Tris‐EDTA. The cross‐linked DNA was then eluted with 1% SDS, 10 mmol/L Tris‐HCl (pH = 8) and 10 mmol/L EDTA at 65°C for 30 minutes. After reversal of formaldehyde cross‐linking, chromatin‐immunoprecipitated DNA samples were treated with RNase A and proteinase K before being harvested for PCR analysis.

### 2.14 Electrophoretic mobility shift assay

Electrophoretic mobility shift assay was performed according to a previous study. 25 Briefly, oligonucleotide probes with a biotin tag at the 5'‐ end of the sequence (Integrated DNA Technologies) were incubated with HEK293T nuclear protein and the working reagent from the Gel shift Chemi‐luminescent EMSA kit (Active Motif 37341). The wild‐type E2F3 EMSA probe sequences were 5'‐GGA ATA CTA ATA AGT CTT AAA AGT TC‐3' and the mutant E2F3 EMSA probe sequences were 5'‐GGA ATC TGC CAA GTC TGC CCA GTT C‐3'. For competitor assays, an unlabelled probe was added to the reaction mixture at 100× excess. The reaction was then incubated for 30 minutes at room temperature and then loaded onto a 6% retardation gel (Thermo Fisher Scientific EC6365BOX) and run in 0.5× TBE buffer. After transfer onto a nylon membrane, the membrane was visualized with the chemiluminescent reagent as recommended. The super shift assay was performed with 1 μg anti‐QKI‐6 antibody (Millipore) and incubated on ice with protein from HEK 293T for 30 minutes prior to addition of oligonucleotide probes and gel electrophoresis.

### 2.15 RNA pull‐down assay

E2F3 3'‐UTR and poly (A)_25_ RNA (100 pmol) were labelled with desthiobiotinylated cytidine bisphosphate using a kit from Thermo Scientific (Waltham, MA). The labelled probes were incubated with samples and isolated using streptavidin magnetic beads in an RNA Capture Buffer for 30 minutes according to the kit protocol. The beads were then washed twice in 20 mmol/L Tris (pH 7.5) buffer and once in the Protein‐RNA Binding Buffer. Next, we added 60 µg of 5637 and T24 extract into the labelled probes and incubated the samples for 45 minutes at 4°C and then washed three times with the Wash Buffer. After 15 minutes incubation with the Biotin Elution Buffer, the samples were eluted and precipitated for Western blot analysis.

### 2.16 Luciferase assay

cDNA fragments with the human *QKI‐6* promoter covering +1 to −2000 bp relative to the transcription initiation site were amplified using PCR primers (Table S1) inserted into a pGL3‐Basic vector (Promega, Madison, WI) and verified by DNA sequencing. For the luciferase assay, HEK‐293T cells were plated in 96‐well plates at a density of 5 × 10^3^ cells/well and grown overnight. Cells were then transfected with 0.5 μg of plasmids carrying the *QKI‐6* promoter along with 0.5 and 1 μg E2F3 expression vectors using Lipofectamine 2000, as well as the pRL construct containing Renilla reniformis luciferase gene, which was used as the normalization control. The luciferase assay was performed with the Dual Luciferase Assay System (Promega) and the relative luciferase activity was defined as the ratio of firefly luciferase activity to *R reniformis* luciferase activity. Error bars represent standard deviation of three experiments.

### 2.17 Statistical analysis

All statistical analyses were performed with spss 17.0 software (SPSS Inc, Chicago, IL). The Pearson chi‐square (χ2) test was used to correlate QKI‐6 expression with clinicopathological data, whereas the Kaplan‐Meier curves and Log rank test were used to analyse overall survival stratified by QKI‐6 expression in bladder cancer patients. For in vitro and nude mouse results, data are expressed as mean ± SD and analysed using a one‐way ANOVA. A *P* < 0.05 was considered statistically significant.

## 3 RESULTS

### 3.1 QKI‐6 down‐regulation in bladder cancer tissues and cell lines

**Figure 1:**
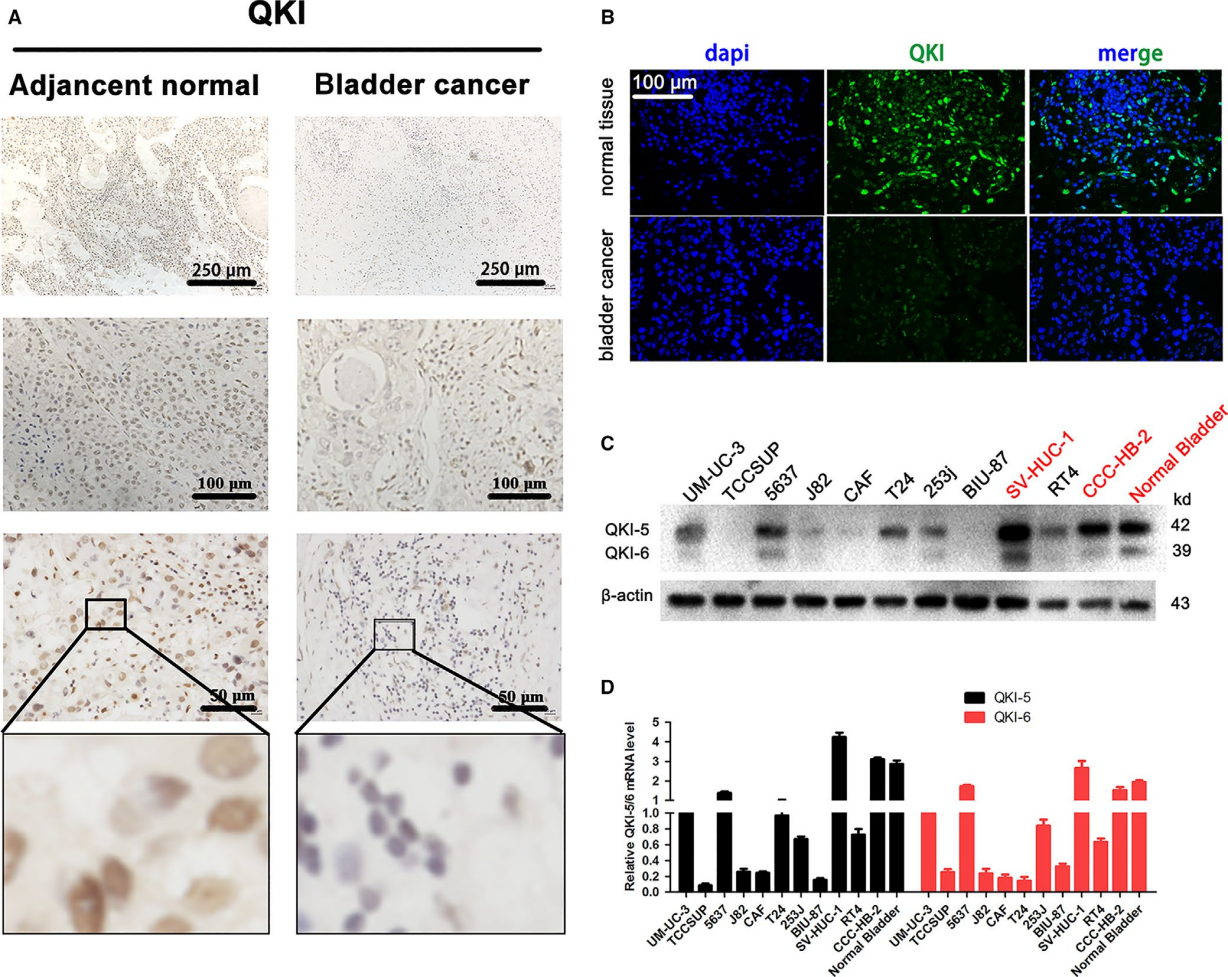
Quaking homologue (QKI) expression is down‐regulated in bladder cancer tissues and cell lines. (A), Immunohistochemistry. QKI protein expression was analysed in bladder cancer and adjacent normal tissues using immunohistochemistry. (B), Immunofluorescence. QKI protein expression was assessed in bladder cancer and adjacent normal tissues using immunofluorescence (200×). (C,D) Western blot and qRT‐PCR. QKI mRNA and protein expression was analysed in different bladder cancer and normal cell lines using Western blot and qRT‐PCR respectively

We first assessed QKI expression in bladder cancer tissues compared to normal tissues and bladder cancer cell lines. We found that QKI‐6 expression was significantly down‐regulated in bladder cancer tissues compared to the matched adjacent normal tissues in 223 patients (Figure 1A,B). Western blot and qRT‐PCR analyses showed that QKI‐6 expression was also lower in bladder cancer cell lines (Figure 1C,D).

### 3.2 Association of QKI‐6 expression with clinicopathological features and survival of bladder cancer patients

**Figure 2:**
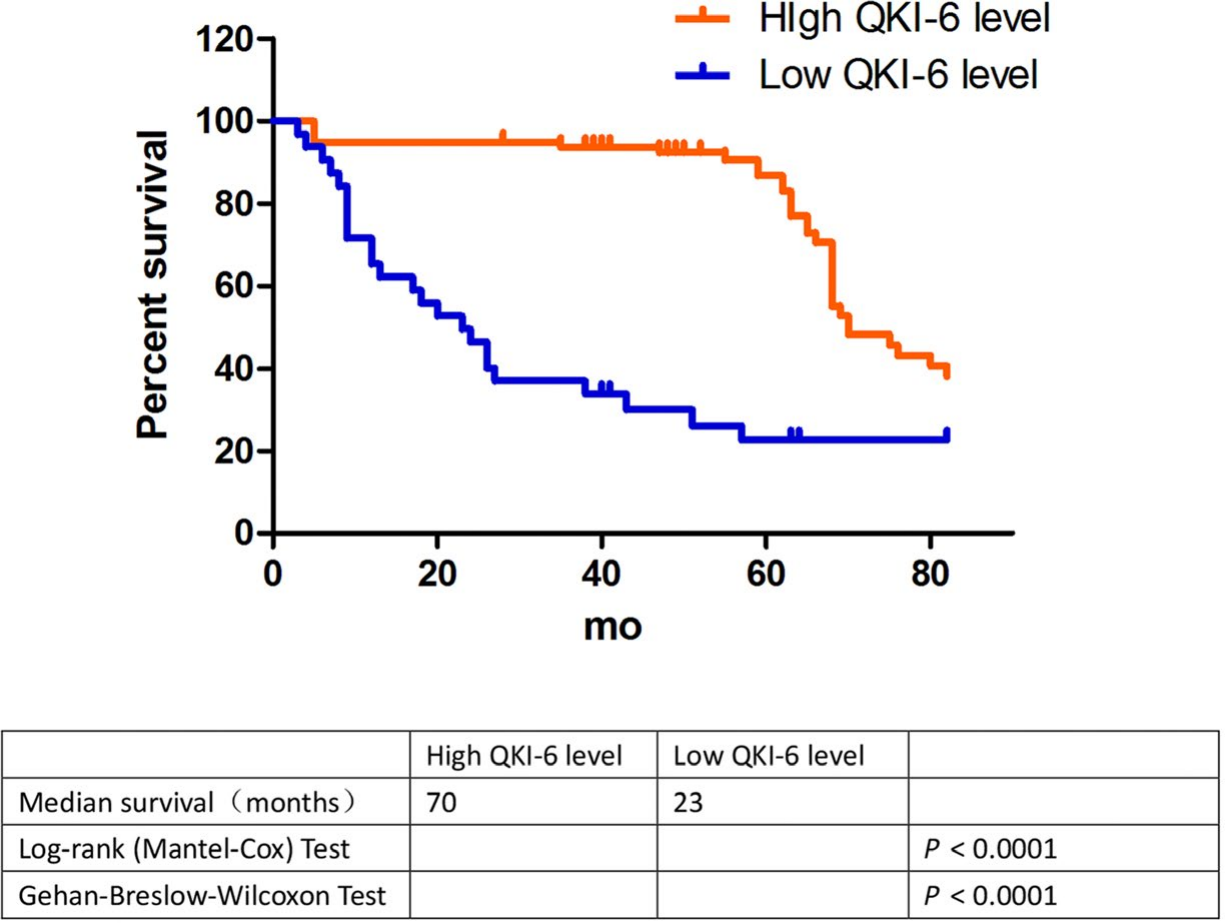
Kaplan‐Meier curves and the log rank analysis of overall survival of bladder cancer patients stratified by quaking homologue (QKI) expression. QKI expression was evaluated in bladder cancer tissue samples with a cut‐off value of ≤30% vs >30% QKI expression, and the overall survival of patients was plotted as Kaplan‐Meier curves and analysed using the log rank test (*P* < 0.0001)

We next assessed whether QKI‐6 expression was associated with clinicopathological features of cancer patients and found that the absence of QKI‐6 expression was associated with advanced pathology stage, tumour TNM stage and depth of invasion (*P* < 0.0001; Table 1), but there was no association with other clinicopathological features, such as age or gender (*P* > 0.05; Table 1). We then correlated QKI‐6 expression with overall patient survival and found that reduced QKI‐6 expression was significantly associated with poor overall survival (Figure 2).

**Table 1:** Association of quaking homolog (QKI) expression with clinicopathological characteristics of bladder cancer patients

Variables	N	QKI expression (n%)		*P* value
		Low (≤30%)	High (>30%)	
Age (y)	223	127 (57)	96 (43)	0.44
≤60	52	32 (62)	20 (38)	
>60	171	95 (56)	76 (44)	
Gender	223	127 (57)	96 (43)	0.20
Male	177	97 (55)	80 (45)	
Female	46	30 (65)	16 (35)	
Pathology stage	223	127 (57)	96 (43)	＜0.0001
I/II	83	31 (37)	52 (63)	
III/IV	140	96 (69)	44 (31)	
pTNM stage	223	127 (57)	96 (43)	＜0.0001
Tis I	47	14 (30)	33 (70)	
II	62	18 (29)	44 (71)	
III	82	66 (80)	16 (20)	
IV	32	29 (90)	3 (10)	
Depth of invasion	223	127 (57)	96 (43)	＜0.0001
Tis T1	62	18 (29)	44 (71)	
T2	52	16 (30)	36 (70)	
T3	89	75 (84)	14 (167)	
T4	20	18 (90)	2 (10)	

### 3.3 QKI‐6 inhibition of bladder cancer cell growth and invasion capacity in vitro

**Figure 3:**
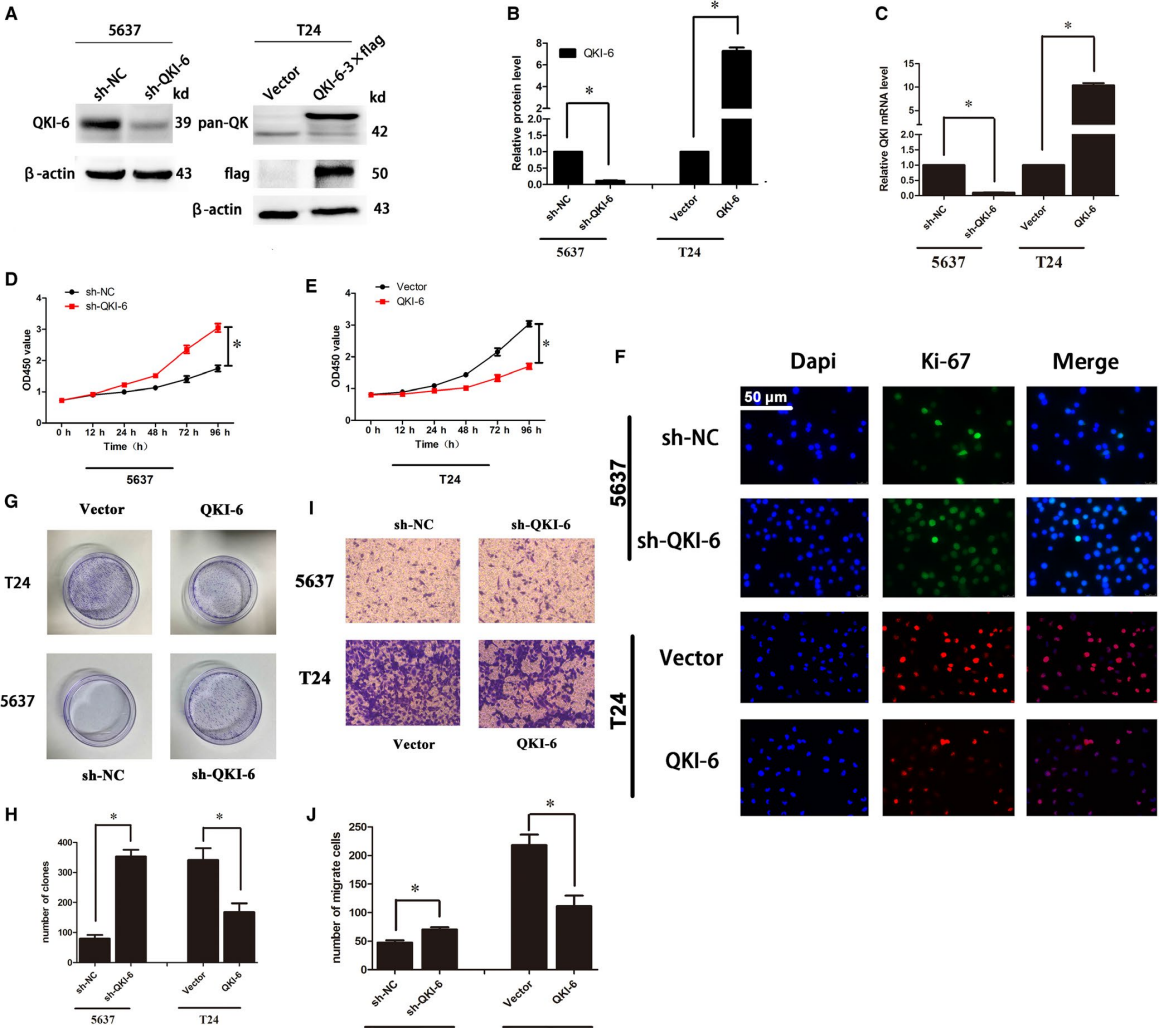
Effects of quaking homologue (QKI)‐6 overexpression and knockdown on regulating bladder cancer cell growth, clone formation and invasion. (A‐C), Western blot and qRT‐PCR. QKI mRNA and protein expression were assessed using Western blot and qRT‐PCR in bladder cancer cell lines 5637 and T24 after infection with lentivirus carrying QKI shRNA or cDNA, respectively. (D‐E), Cell viability CCK‐8 assay. Bladder cancer cell lines 5637 and T24 were infected with lentivirus carrying QKI shRNA, QKI cDNA or their negative controls, respectively, and then subjected to CCK‐8 assay. Cell growth curves are based on average absorbance values (n = 6). (F), Immunofluorescence. Bladder cancer cell lines 5637 and T24 were infected with lentivirus carrying QKI shRNA, QKI cDNA or their negative controls, respectively, and then subjected to immunofluorescence analysis of Ki‐67 expression (400×). (G), Colony formation assay. Duplicated cells were subjected to the tumour cell colony formation assay. (H), Quantified data of G. (I), Transwell invasion assay. Bladder cancer cell lines 5637 and T24 were infected with lentivirus carrying QKI shRNA, QKI cDNA or their negative controls, respectively, and then subjected to Transwell invasion assay (400×). (J), Quantified data of I. All assays were performed at least three times independently and error bars represent the mean ± SD and one‐way ANOVA analysis of three independent experiments. *P < 0.05

**Figure 4:**
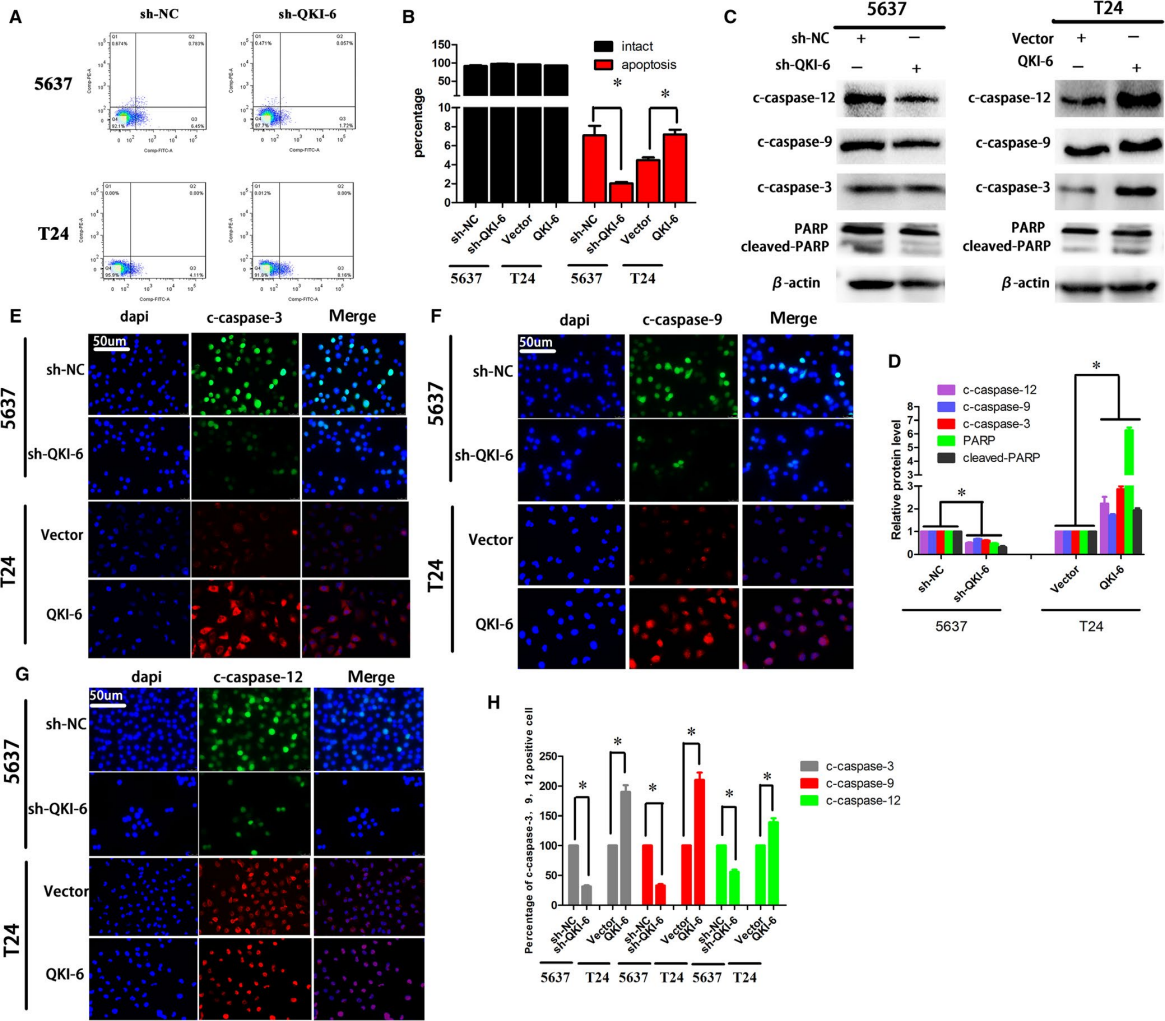
Quaking homolog (QKI)‐6 induced bladder cancer cell apoptosis in vitro. (A), Flow cytometric apoptosis assay. Bladder cancer cell lines 5637 and T24 were infected with lentivirus carrying QKI shRNA, QKI cDNA or their negative controls, respectively, and then subjected to flow cytometric analysis. (B), Quantified data of A. (C), Western blot. Duplicated cells were subjected to Western blot analysis of cleaved caspase‐12, caspase‐9 and caspase‐3, and PARP expression. (D), ImageJ software quantitation of Western blot from C. (E‐G), Immunofluorescence. Bladder cancer cell lines 5637 and T24 were infected with lentivirus carrying QKI shRNA, QKI cDNA or their negative controls, respectively, and then subjected to immunofluorescence analysis of cleaved caspase‐12, caspase‐9 and caspase‐3 (400×). (H), ImageJ software quantitation of E‐G. Data are presented as the mean ± SD following one‐way ANOVA analysis for three independent experiments. *P < 0.05

**Figure 5:**
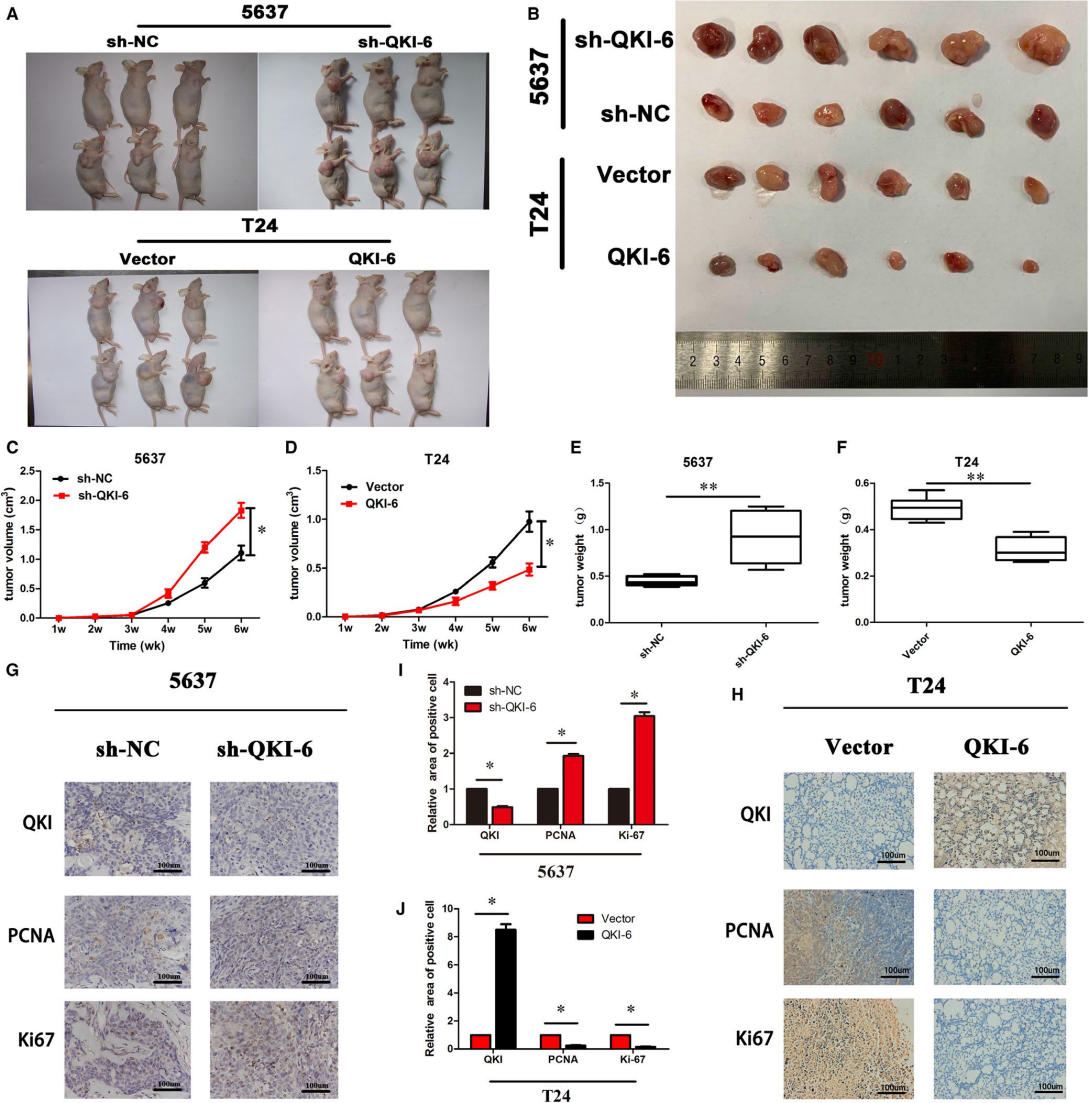
Anti‐tumour activity of quaking homolog (QKI)‐6 in nude mouse tumour cell xenografts. (A,B), Nude mice and tumour cell xenografts. Representative subcutaneous tumour cell xenografts were obtained from nude mice that were injected with 1 × 10^6^ cells for 2 wk and sacrificed thereafter. (C, D), Tumour volumes from each group (n = 6). **P < 0.05 vs the control. (E,F), Tumour weights (n = 6). *P < 0.05 vs the control. (G,H) Immunohistochemistry. Tumour cell xenografts were assessed using immunohistochemistry to detect expression of PCNA and Ki‐67. (I,J) Quantified data of G and H, respectively, as the mean ± SD. *P < 0.05 vs the control

**Figure 6:**
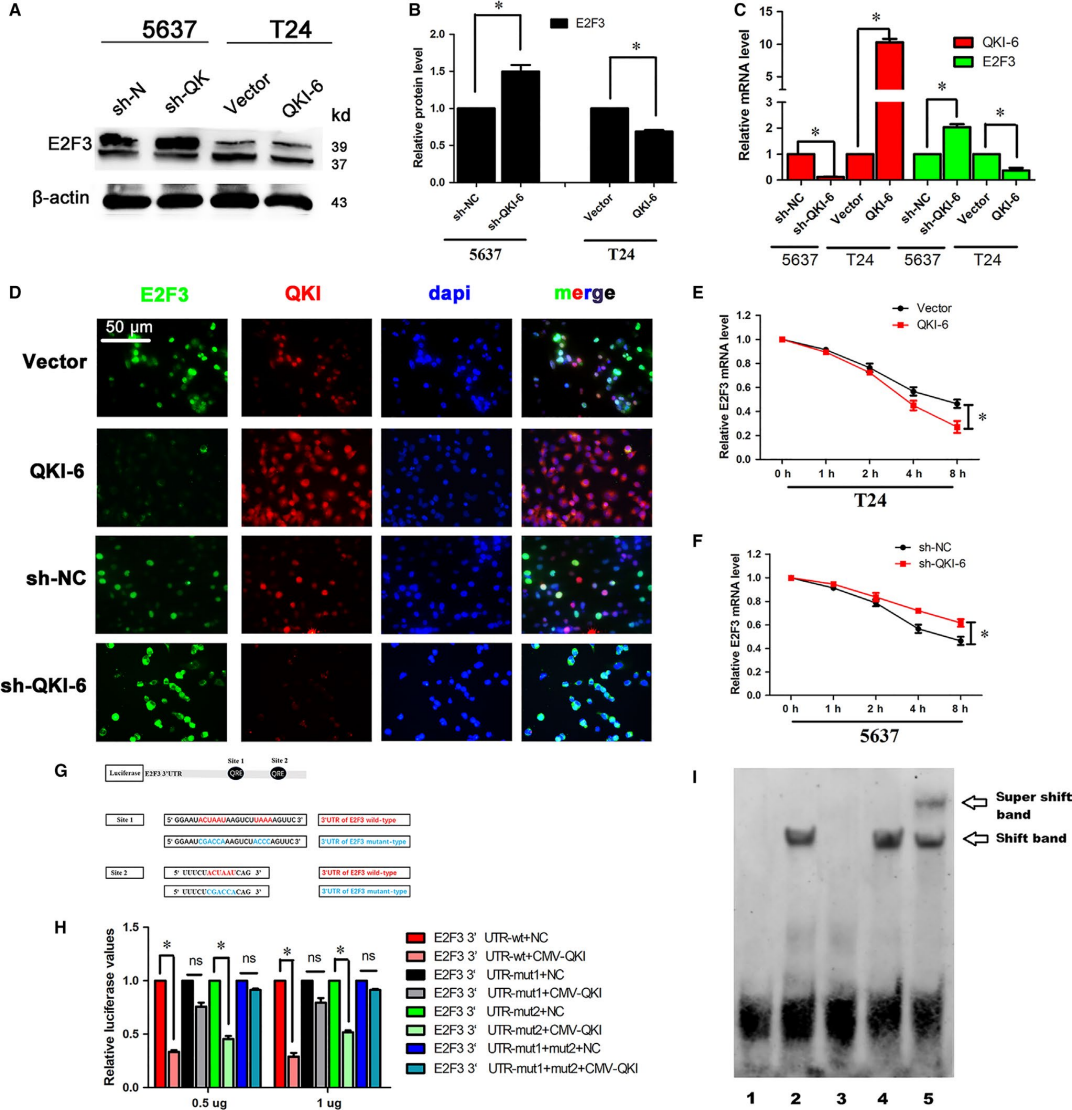
Quaking homologue (QKI)‐6 regulates E2 transcription factor 3 (E2F3) expression via directly binding to the E2F3 3'‐UTR. (A), Western blot. Bladder cancer cell lines 5637 and T24 were infected with lentivirus carrying QKI‐6 shRNA, QKI‐6 cDNA or their negative controls, respectively, and then subjected to Western blot. (B), Quantified data of A. (C), qRT‐PCR. Bladder cancer cell lines 5637 and T24 were infected with lentivirus carrying QKI‐6 shRNA, QKI‐6 cDNA or their negative controls, respectively, and then subjected to qRT‐PCR analysis of QKI and E2F3. (D), Double‐immunofluorescence labelling showed that QKI‐6 co‐localized with E2F3 and restrained E2F3 expression in both the nuclei and cytoplasm. (E,F) qRT‐PCR. Bladder cancer cell lines 5637 and T24 were infected with lentivirus carrying QKI‐6 shRNA, QKI‐6 cDNA or their negative controls, respectively, for 24 h and then treated with 5 mg/mL actinomycin D for 1, 2, 4 and 8 h and then subjected to qRT‐PCR analysis of E2F3 mRNA. The data are representative of three independent experiments. (G), Scheme of constructs used for the dual luciferase assays. The position of the putative QRE sequence in the E2F3 3'‐UTR is depicted as a black cycle. Sequences of the mutated QKI response element (QRE) are shown below. (H), Luciferase activity assay. HEK‐293T cells were grown and transiently transfected with pGL3‐E2F3 3'‐UTR wild‐type, pGL3‐E2F3 3'‐UTR mutation reporter or the internal control vector pGL‐3 for 24 h and then subjected to the luciferase reporter assay. The fold changes were calculated and expressed as means ± SD (n = 3). *P < 0.05. (I), Gel sift assay. Electrophoretic mobility shift assays (EMSAs) of E2F3 RNAs with QKI‐6‐flag or with the buffer alone. The RNA used for the EMSA is the E2F3 QRE‐1. Migration patterns of unbound RNAs (free probe) and protein/bound RNAs (QKI/RNA complex) are indicated on the right. Lane 1, negative control; lane 2, sample; lane 3, cold probe competition; lane 4, mutant cold probe competition; and lane 5, the super‐shift

**Figure 7:**
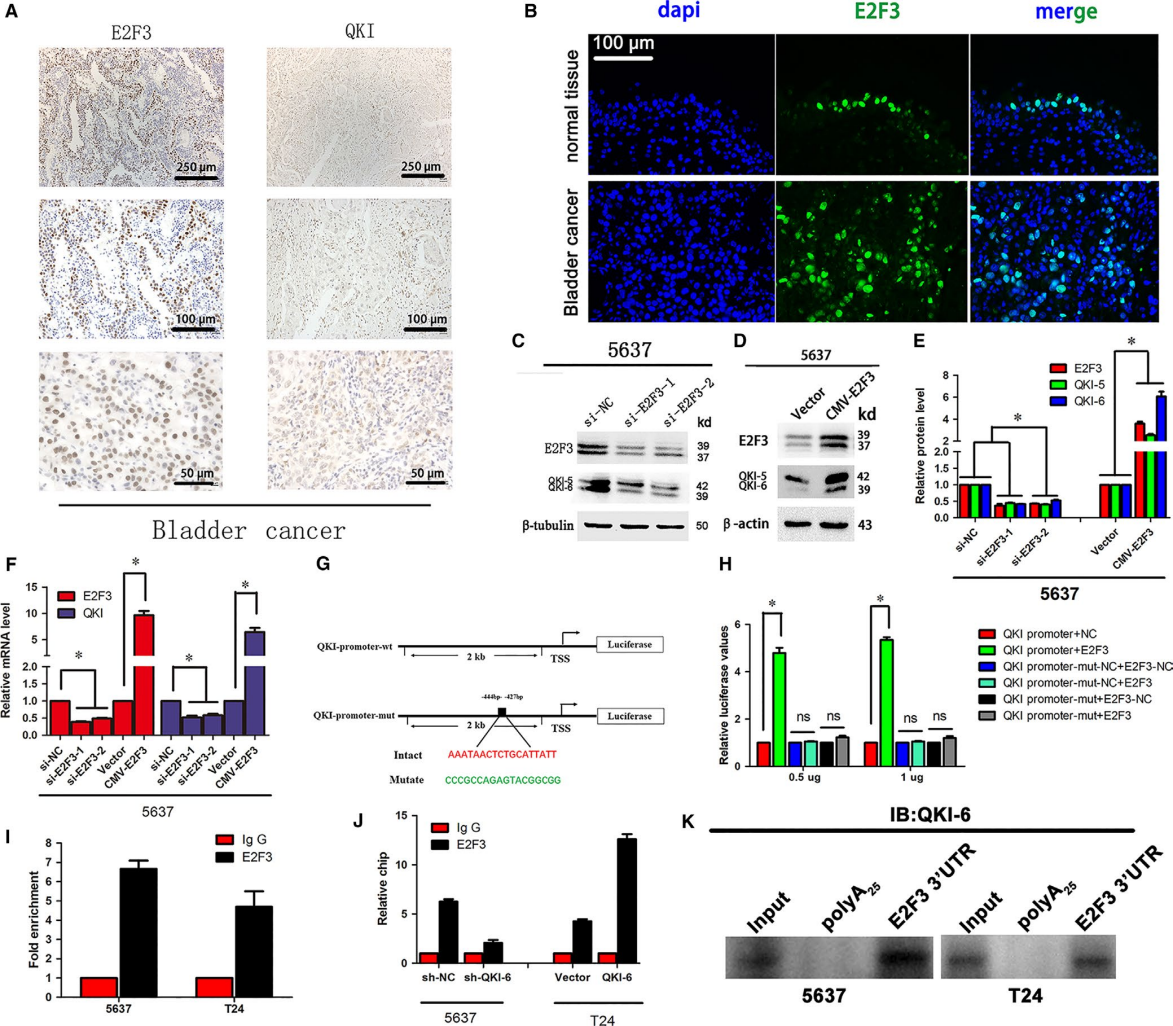
E2 transcription factor 3 (E2F3) regulates quaking homologue (QKI) expression in bladder cancer cells in vitro and ex vivo. (A) Immunohistochemistry. Bladder cancer tissues were immunostained with E2F3 and QKI antibodies. (B) Immunofluorescence. Bladder cancer tissues were immunostained with E2F3 and QKI antibodies. (200x). (C,D) Western blot. Bladder cancer cell lines 5637 and T24 were infected with lentivirus carrying QKI‐6 shRNA, QKI‐6 cDNA or their negative controls, respectively, and then subjected to Western blot. (E) Quantified data of D. (F) qRT‐PCR. Bladder cancer cell lines 5637 and T24 were infected with lentivirus carrying QKI‐6 shRNA, QKI‐6 cDNA or their negative control, respectively, and then subjected to qRT‐PCR. (G) Scheme of constructs used for the dual luciferase assay. (H) Luciferase reporter assay. HEK‐293T cells were grown and transiently transfected with pGL3‐QKI promoter wild‐type or mutant reporter, internal control vector pGL‐3 for 24 h and then subjected to the luciferase reporter assay. (I and J) chromatin immunoprecipitation (ChIP) assay. T24 and 5637 cells were infected with (J) or without (I) lentivirus before ChIP assay. ChIP analysis was run using either the E2F3 or IgG antibody. (K) Western blot analysis of QKI‐6 in precipitates isolated from 5637 and T24 cell lysates using in vitro desthiobiotin‐labelled E2F3 3'‐UTR or poly (A)_25_ RNA labelling. Results are presented as the mean ± SD. *P < 0.05 vs the control

**Figure 8:**
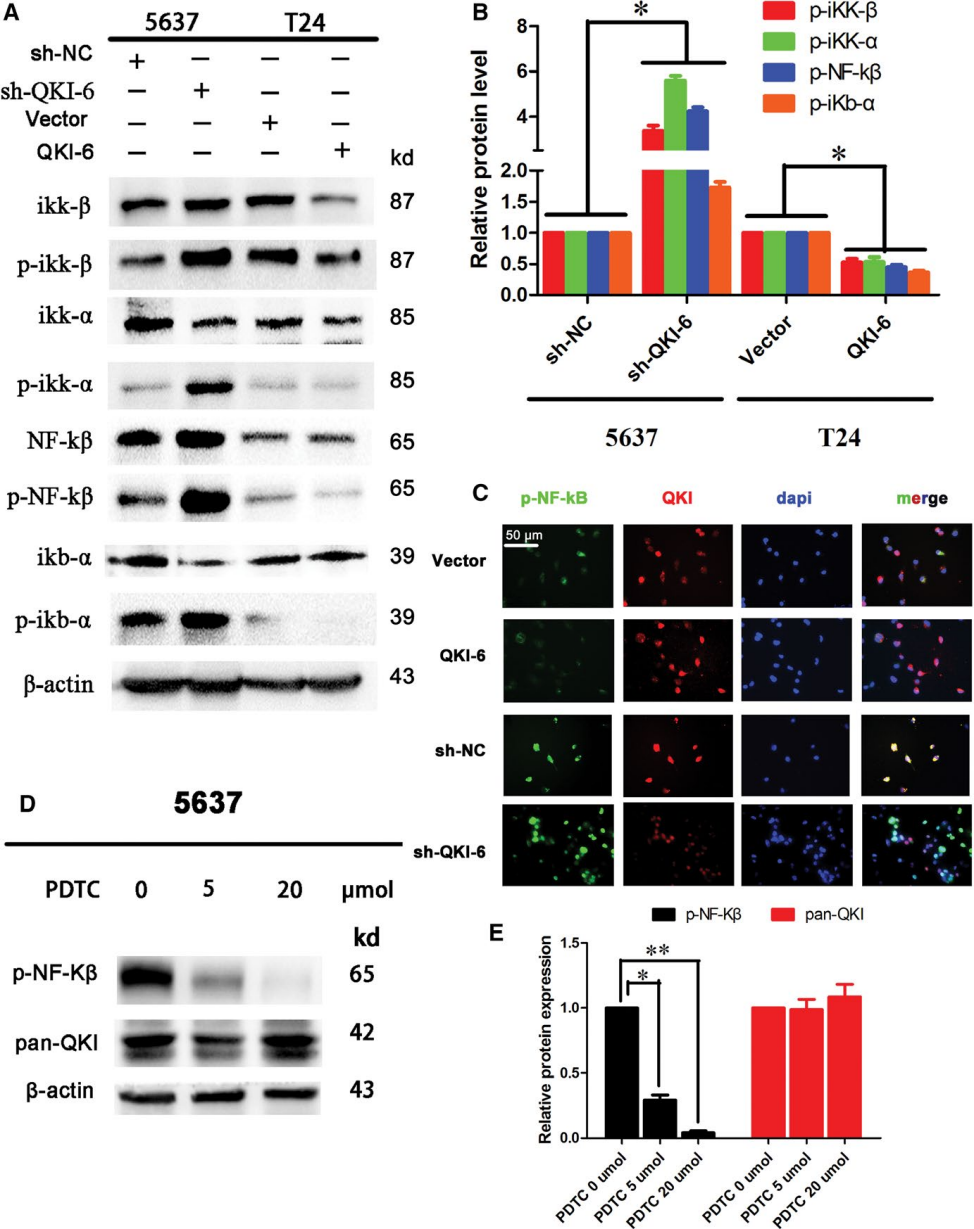
Quaking homologue (QKI)‐6 suppresses NF‐κB signalling in bladder cancer cells. (A) Western blot. Bladder cancer cell lines 5637 and T24 were infected with lentivirus carrying QKI‐6 shRNA, QKI‐6 cDNA or their negative controls, respectively, and then subjected to Western blot analysis of ikk‐β, p‐ikk‐β, ikk‐α, p‐ikk‐α, NF‐κβ, p‐NF‐κβ, ikb‐α and p‐ikb‐α. (B) Quantified data of A. (C) Double‐immunofluorescence labelling showed that QKI‐6 co‐localized with p‐NF‐κB and restrained p‐NF‐κB translocation in the nucleus (400×). (D) Western blot showed that p‐NF‐κB expression was reduced after pyrrolidinedithiocarbamate (PDTC) treatment at both 5 and 20 µmol. (E) Quantified data of D

**Figure 9:**
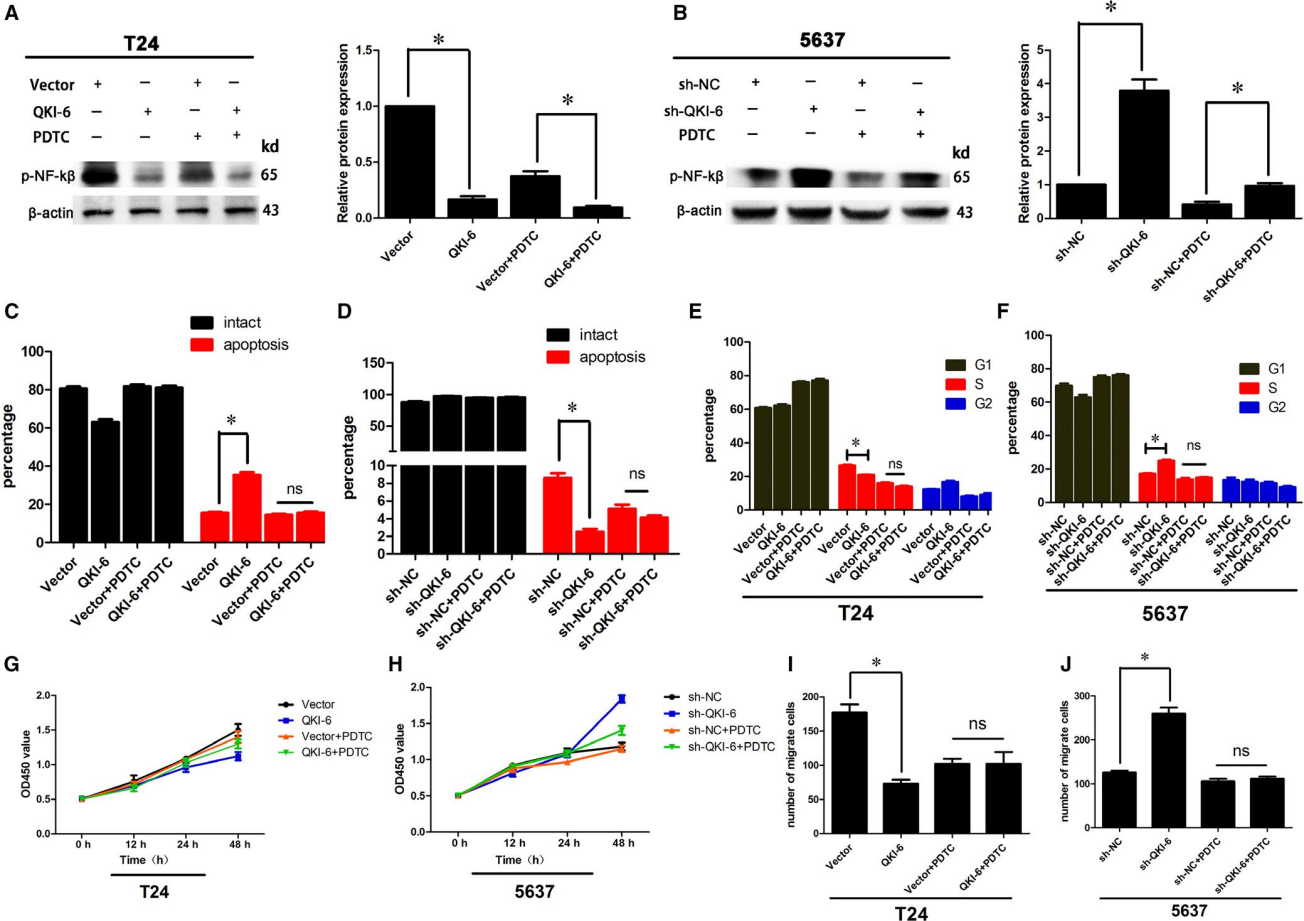
Effects of the NF‐κB inhibitor pyrrolidinedithiocarbamate (PDTC) on quaking homolog (QKI)‐6‐induced cell apoptosis, cell cycle arrest, cell proliferation and invasion. (A,B) Western blot showed that PDTC significantly inhibited p‐NF‐κB in both QKI‐6 overexpressing T24 and QKI‐6 knockdown 5637 (sh‐QKI‐6) cells. (C,D) QKI‐6‐induced cell apoptosis was abolished after addition of the NF‐κB inhibitor PDTC in T24 and 5637 cells. (E,F) The number of tumour cells in the cell cycle S phase was similar in both vector and QKI‐6 cDNA‐transfected T24 cells after addition of PDTC. The same trend was observed in the sh‐NC and sh‐QKI‐6‐tranfected 5637 cells. (G,H) Cell proliferation was significantly decreased by the QKI‐6 plasmid and enhanced by the sh‐QKI‐6 plasmid, whereas there was no significant difference after PDTC treatment. (I,J) Invasion capacity was also abolished by PDTC treatment in T24 and 5637 cells. Each assay was repeated at least three times. *P < 0.05

**Figure 10:**
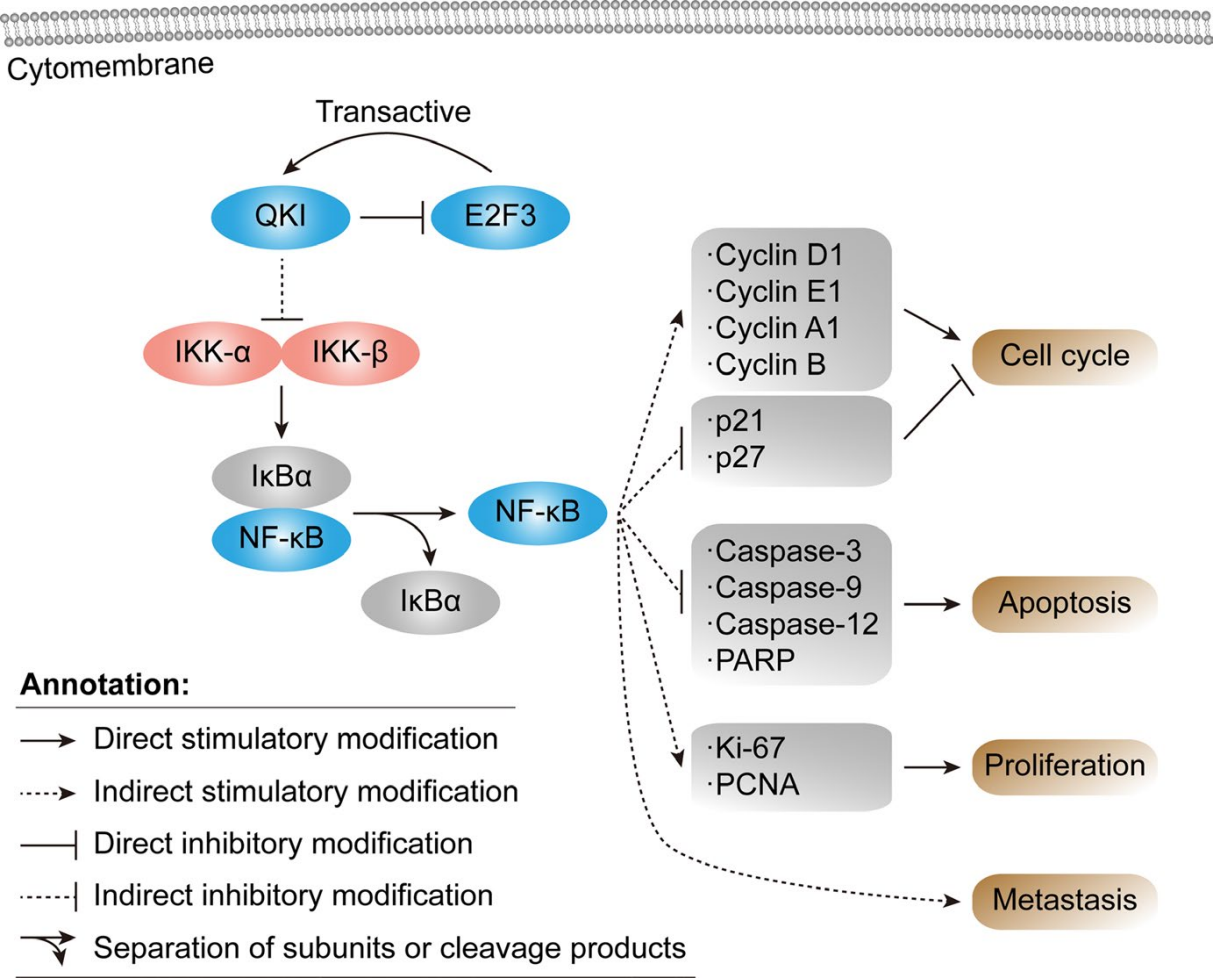
Schematic illustration of the quaking homolog (QKI)‐6 inhibited bladder cancer progression. Schematic representation of the function of QKI in coordinately targeting multiple cell cycle, cell apoptosis, cell differentiation and cell proliferation regulators in bladder cancer. QKI‐6 targets the NF‐κβ signalling pathways, which, subsequently results in cell cycle arrest, cell differentiation, cell apoptosis, decreased proliferation. Activated E2 transcription factor 3 (E2F3) transcribes QKI, which represses the activity of E2F3

Our current ex vivo data demonstrated that QKI‐6 expression was reduced in bladder cancer tissues and cells and that QKI‐6 down‐regulation was associated with poor overall survival. We thus further assessed if changes in QKI‐6 expression affect bladder cancer cell malignant behaviours. Western blot data showed that bladder cancer cells expressed different levels of QKI‐6 proteins (Figure 3). We chose 5637 for sh‐QKI‐6 and T24 for QKI‐6 cDNA transfections. Our data showed that QKI‐6 cDNA up‐regulated QKI‐6 expression, whereas QKI‐6 shRNA reduced QKI‐6 expression in T24 and 5637 cells (Figure 3, 4, 5, 6, 7, 8, 9, 10A‐C and Figure S1A). Immunofluorescence showed that QKI‐6 expression was up‐regulated in T24 cells and down‐regulated in 5637 cells (Figure S1B,C). We then assessed the effects of QKI‐6 up‐ and down‐regulation on bladder cancer cell proliferation and found that down‐regulation of QKI‐6 expression promoted cell proliferation (Figure 3D), whereas up‐regulation of QKI‐6 expression inhibited tumour cell proliferation in 5637 cells (Figure 3E). Immunofluorescence showed that overexpression of QKI‐6 reduced expression of the cell proliferation marker, Ki67 (Figure 3F). Tumour cell colony formation and Transwell invasion assays further showed that QKI‐6 overexpression reduced the number of tumour cell colonies (Figure 3G,H) and inhibited tumour cell invasion (Figure 3I,J), respectively.

### 3.4 QKI‐6 promotion of tumour cell apoptosis and cell cycle arrest in vitro

Next, we determined the effects of QKI‐6 overexpression or knockdown on tumour cell apoptosis and cell cycle distribution. We found that the percentage of apoptotic 5637 cells was significantly lower after QKI‐6 knockdown, whereas the percentage of apoptotic T24 cells was significantly higher after QKI‐6 cDNA transfection compared to control cells (Figure 4A,B). Western blot data further showed that expression of caspase‐3, caspase‐9 caspase‐12, and PARP was up‐ or down‐regulated in these 5637 and T24 cells respectively (Figure 4C,D), which was also confirmed by immunofluorescence (Figure 4E‐H). Moreover, QKI‐6 knockdown and up‐regulation in 5637 and T24 cells induced and reduced, respectively, the proportion of cells in S phase compared to the control group (Figure S2A,B). Western blot data showed that cyclin D1, cyclin B, cyclin E1 and cyclin A1 expression were up‐regulated, whereas levels of p27 and p21 expression were down‐regulated in QKI‐6‐knocked down 5637 cells compared to the negative control cells. In contrast, QKI‐6 overexpression in T24 cells inversely affected the tumour cells (Figure S2C,D). These findings suggest that QKI‐6 expression promotes cell apoptosis and induces cell cycle arrest in the G2/M phase of the cell cycle.

### 3.5 Anti‐tumour activity of QKI‐6 in vivo

To confirm the inhibitory effects of QKI in vivo, we infected bladder cancer 5637 cells with shRNA‐NC or shRNA‐QKI‐6 lentivirus particles and T24 cells with lentivirus particles carrying vector only or QKI‐6 cDNA. Transfection efficiency was verified, as shown in Figure 3A. We then subcutaneously injected these cells into nude mice and monitored tumour cell xenograft formation and growth. We found a significant difference in tumour cell xenograft formation growth and weight (Figure 5). Specifically, the size of tumour xenografts was larger in the shRNA‐QKI‐6 5637 cells compared to the negative control shRNA xenografts, whereas the tumour xenografts were consistently smaller in vector‐only T24 cells compared to those of CMV‐QKI‐6 T24 cells (Figure 5A,B). Immunohistochemical data showed that PCNA and Ki‐67 expression were significantly up‐regulated following QKI‐6 knockdown in 5637 cells compared to control cells (Figure 5G,I). However, PCNA and Ki‐67 expression were reduced in tumour cell xenografts injected with QKI‐6 overexpressing T24 cells (Figure 5H,J). Immunofluorescence staining of tissue samples showed down‐regulation of Ki‐67 and PCNA levels but up‐regulation of PARP levels in T24 QKI‐6 overexpressing tumour cell xenografts, whereas knockdown of QKI‐6 expression had the opposite effects (Figure S3A‐E). These results suggest that QKI‐6 suppresses tumour growth in vivo.

### 3.6 QKI reduces E2F3 mRNA stability by directly binding to the E2F3 3'‐untranslated region

As an RNA‐binding protein, QKI post‐transcriptionally regulates expression of target mRNAs.^15^, ^18^ We performed a bioinformatic analysis and found two potential QREs in the E2F3 3'‐untranslated region (3'‐UTR). We thereafter assessed whether QKI‐6 overexpression or knockdown affects E2F3 expression using Western blot and qRT‐PCR analyses (Figure 6, 7, 8, 9, 10A‐C). Double‐immunofluorescence labelling showed that QKI‐6 co‐localized with E2F3 and inhibited E2F3 expression and nuclear translocation (Figure 6D). Our data showed that QKI‐6 overexpression or knockdown modulated the stability of E2F3 mRNA after cells were treated with actinomycin D, an inhibitor of gene transcription (Figure 6E,F). To further validate whether QKI‐6 directly regulates E2F3, we constructed plasmids carrying the wild‐type or mutant E2F3 3'‐UTR (Figure 6G) and performed a dual luciferase assay. We found that QKI‐6 overexpression reduced the luciferase activity compared to the mutated controls (Figure 6H). Furthermore, EMSA demonstrated that QKI‐6 bound to the E2F3 mRNA and generated a super‐shift band (Figure 6I). These results suggest that QKI‐6 decreases stability of E2F3 mRNA by directly binding to the E2F3 3'‐UTR.

### 3.7 E2F3 modulation of QKI‐6 transcription by binding to its promoter

As a transcription factor, E2F3 has an established role in regulating cell cycle progression. A recent study reported that amplification and overexpression of E2F3 resulted in oncogenic activity in human bladder cancer development.^26^ E2F3 expression can be regulated by binding of promoter regions in target genes.^27^ We therefore performed immunohistochemistry and immunofluorescence analyses and found that QKI‐6 expression was inversely associated with E2F3 expression in bladder cancer tissue specimens (Figure 7A,B), whereas E2F3 expression was inversely associated the QKI‐6 mRNA and protein expression (Figure 7, 8, 9, 10C‐F).

Furthermore, we cloned the QKI‐6 promoter region (−2000 to +1 bp) containing the E2F3‐binding site and the mutant gene without the E2F3‐binding site (Figure 7G). We found that E2F3 enhanced QKI‐6 promoter luciferase activity (Figure 7H). The Chromatin immunoprecipitation (ChIP) assay also demonstrated that E2F3 directly binds to the QKI‐6 promoter (Figure 7I). This effect was more obvious in QKI‐6 up‐regulated T24 cells, but the luciferase activity was reduced in QKI‐6 down‐regulated 5637 cells (Figure 7J). Our RNA pull‐down assay of 5637 and T24 cell lysates with E2F3 3'‐ UTR showed a specific interaction with QKI‐6 (Figure 7K). These results illustrate that E2F3 directly affects transcription of QKI‐6, forming a negative feedback loop.

### 3.8 **QKI‐6 inhibits activity and expression of NF‐**κ**B signalling proteins**

Transactivation of NF‐κB can be initiated by a vast array of stimuli that have different biological activities, such as inflammation, immunity, differentiation, cell growth, tumorigenesis and apoptosis.^28^, ^29^, ^30^ A previous study reported that NF‐κB was frequently activated in bladder cancer.^31^, ^32^ We also have preliminary evidence demonstrating that QKI‐6 inhibits bladder tumorigenesis (data not shown). In this study, we measured NF‐κB expression and activity in QKI‐6‐knockdown or overexpressing bladder cancer cells. We found that QKI‐6 knockdown in 5637 cells up‐regulated the expression of phosphorylated (p)‐NF‐κβ, Ikk‐α, Ikk‐β and Ikb‐α, whereas QKI‐6 overexpression in T24 cells reduced expression of all of these proteins (Figure 8A,B). Double‐labelling immunofluorescence staining showed that QKI‐6 co‐localized with p‐NF‐κB and inhibited p‐NF‐κB expression and nuclear translocation (Figure 8C). The expression of p‐NF‐κB was significantly inhibited by different concentrations of PDTC, whereas QKI was not affected (Figure 8D,E). This result demonstrates that QKI is upstream of NF‐κB and that QKI down‐regulates NF‐κB pathway activation.

### 3.9 **Effects of the NF‐**κ**B inhibitor PDTC on regulating tumour cell proliferation, apoptosis, cell cycle arrest and invasion**

We found that 20 µmol of PDTC treatment for 6 hours significantly down‐regulated p‐NF‐κB in both the T24 vector and QKI‐6 group and in the 5637 sh‐NC and sh‐QKI‐6 group (Figure 9A,B). However, we found that tumour cell apoptosis in these QKI‐6‐altered T24 and 5637 cells was not significantly different (Figure 9C,D and Figure S4A,B), nor were there any significant differences in the cell cycle distributions in QKI‐6 overexpressing or knockdown T24 and 5637 cells (Figure 9, 10E,F and Figure 4C,D). These findings indicate that PDTC treatment abolished the effects of QKI‐6 on these bladder cancer cells. Although cell proliferation was reduced after QKI‐6 overexpression and knockdown, addition of PDTC showed that the OD450 value was not statistically different between the vector and QKI‐6 cDNA‐transfected T24 cells or between the sh‐NC and sh‐QKI‐6‐transfected 5637 cells (Figure 9G,H), also indicting that PDTC treatment abolished the effects of QKI‐6 on these bladder cancer cells. In addition, although tumour cell invasion capacity was impaired after both QKI‐6 overexpression and knockdown, PDTC had no significant effect (Figure 9I,J and Figure S4E,F). The schematic illustration of the QKI‐6 inhibited bladder cancer progression was shown in Figure 10. Thus, these results suggest that QKI‐6 plays a tumour suppressor role through inactivating the NF‐κB signalling pathway in bladder cancer cells.

## 4 DISCUSSION

Recently, altered QKI expression was implicated in various human cancers, including colorectal cancer,^33^ paediatric brain tumours,^34^ glioma,^35^ renal clear cell carcinoma,^9^ lung cancer^17^ and prostate cancer.^18^ In this study, we measured QKI‐6 expression and determined its role in regulating bladder cancer in vitro and in vivo, and we also investigated the molecular mechanism underlying QKI‐6’s effect in bladder cancer cells. We found that QKI‐6 expression was reduced in bladder cancer tissues and that QKI‐6 down‐regulation was associated with advanced bladder cancer TNM stage and poor patient overall survival. We also revealed that QKI‐6 inhibits bladder cancer cell growth and invasion capacity, but induces tumour cell apoptosis and cell cycle arrest in vitro. The nude mouse xenograft model confirmed QKI‐6’s anti‐tumour activity in vivo, as evidenced by reduced tumour xenograft growth and increased expression of Ki67 and PCNA. In contrast, QKI‐6 knockdown had the opposite effect on bladder cancer cells in vitro and in vivo. In addition, QKI‐6 decreased E2F3 expression by directly binding to the E2F3 3'‐UTR, whereas E2F3 promoted transcription of QKI‐6 mRNA by binding to the *QKI*‐6 promoter, resulting in a negative feedback loop, which likely stabilizes both E2F3 and QKI‐6 levels. QKI‐6 overexpression also reduced the activity and expression of NF‐κB signalling proteins in vitro*,* implying a multi‐faceted regulatory network of QKI‐6 with downstream molecular and signalling pathways. Taken together, our current data demonstrate that QKI‐6 expression is down‐regulated in bladder cancer development and progression. Future studies will investigate if targeting QKI‐6 could be a novel strategy to control bladder cancer.

Initially, QKI was identified as an RNA‐binding protein known to be important in the central nervous system and peripheral nervous system, where it regulates mRNA stability, translation, splicing and cytoplasmic/nuclear localization of various target genes.^36^ More recent studies have shown that QKI possesses anti‐tumour activity; for example QKI can induce cell cycle arrest and apoptosis, as well as inhibit growth, invasion and clone formation of colon cancer, oral cancer and renal cell carcinoma.^9^, ^11^, ^16^, ^18^ A previous study showed that QKI down‐regulation was associated with poor prognosis of gastric cancer,^14^ which supports the findings presented here. Our current study also demonstrated that QKI‐6 expression is associated with advanced tumour stages of bladder cancer patients, which further validated previous in vitro data showing that QKI suppresses colon and oral tumorigenicity^11^, ^16^ and prostate cancer.^18^ Although there are several QKI isoforms,^9^, ^10^ our current findings showed that QKI‐6 was significantly down‐regulated in bladder cancer tissues and cell lines compared to other QKI isoforms.

To assess the effects of QKI‐6 in bladder cancer, we knocked down and overexpressed QKI‐6 in bladder cancer cells and then conducted cell viability CCK‐8, Transwell and colony formation assays. Our data showed that QKI‐6 overexpression reduced T24 cell proliferation but promoted tumour cell arrest at the G2/M phase of the cell cycle and decreased the S phase cell population, as well as impaired T24 cell invasion ability. In contrast, QKI‐6 knockdown inversely affected the bladder cancer 5637 cells. Although QKI‐6 data in human cancer are limited, our current data further support a role for QKI‐6 in different human cancers.^9^, ^18^, ^35^ Our current study in bladder cancer using both ex vivo and in vitro approaches is novel, as our data indicate that QKI‐6 plays a tumour suppressive role in bladder cancer.

Functionally, QKI‐6 can regulate expression of various target genes at the post‐transcriptional level.^8^ QKI‐6 can regulate gene expression via directly binding to target 3'‐UTRs containing QRE sites^12^; for example many genes that are related to cell cycle progression, cell differentiation or apoptosis have been identified as QKI targets. Specifically, β‐catenin, p27, p53, cyclinD1, cyclinE1, E2F1 and others are QKI gene targets.^11^, ^13^ In our current study, we found that E2F3 is a novel QKI‐6 target gene. E2F3 has been shown to promote cell cycle progression and potentially play an oncogenic role in different human cancers, like bladder cancer.^26^, ^37^ Our current study showed an inverse association between QKI‐6 and E2F3 expression in bladder cancer tissues and cells. We also found that QKI‐6 could post‐transcriptionally down‐regulate E2F3 expression, whereas E2F3 promoted QKI‐6 expression in bladder cancer cells, indicating a negative feedback mechanism in gene regulation. The QKI‐6 and E2F3 feedback loop could potentially and synergistically maintain each other's expression in bladder cancer. Indeed, a previous study demonstrated that QKI interacted with E2F1 in a negative feedback loop to regulate cell cycle distribution.^13^ Other previous studies revealed that QKI could either increase or decrease the stability of target mRNAs.^13^, ^16^, ^38^ In our current study, we treated bladder cancer cells after QKI‐6 knockdown or overexpression with a gene transcription inhibitor, actinomycin D, and found that QKI‐6 destabilized and reduced E2F3 mRNA and protein respectively. Furthermore, our EMSA and ChIP assays confirmed the direct binding of QKI‐6 to the E2F3 mRNA 3'‐UTR. Taken together, our current data revealed that QKI‐6 can directly regulate E2F3, whereas QKI‐6 itself was also regulated by E2F3, suggesting that QKI‐6 and E2F3 form a negative feedback loop. However, the underlying mechanism of this feedback loop in bladder cancer progression requires further investigation.

A previous study reported that QKI deficiency induced inflammation in experimental endotoxemia via increasing NF‐κB signalling,^39^ whereas another recent study showed that NF‐κB repression of QKI expression promotes cancer stem cell‐like properties during the neoplastic transformation of hepatic cells in response to arsenite.^40^ These two studies clearly indicate that reduced QKI‐6 expression in bladder cancer tissues or cells activates the NF‐κB signal pathway, which might play a key role in cancer initiation, especially in bladder cancer.^28^, ^29^ Our current data showed that up‐regulated QKI‐6 expression reduced the activity and expression of NF‐κB signalling proteins in bladder cancer.^31^, ^32^

In conclusion, we demonstrated that QKI‐6 down‐regulation in bladder cancer tissues and cells promoted tumour cell malignant behaviours by down‐regulating E2F3 and NF‐κB signalling pathways (Figure 9). In contrast, QKI‐6 overexpression inhibited bladder cancer cell proliferation, cell cycle progression and invasion, but promoted cell apoptosis and cell cycle arrest at the G2/M phase. Furthermore, reduced QKI‐6 expression was associated with advanced bladder cancer TNM stage and poor survival of patients. Future studies are needed to clarify the specific mechanism by which QKI‐6 regulates E2F3 and NF‐κB signalling in bladder cancer development and progression. Specifically, it would be important to investigate if restoring QKI‐6 expression could be a novel strategy to restrict bladder cancer.

## CONFLICT OF INTEREST

The authors declare that they have no conflict of interest.

## AUTHOR CONTRIBUTIONS

FS, ZD, ZZ, SJX and BMH designed the study. XYB, BYY, QS, XJW and QW collected and analysed the data. CYJ and RZZ advised on histological staining and analysis. FS, DC contributed samples collection and intellectual input. FS, ZD drafted and wrote the manuscript. SJX and BMH revised the manuscript critically for intellectual content. All authors provided intellectual input and approved the final version of the manuscript.

## Data Availability

The datasets generated and analysed during this study are available from the corresponding author on reasonable request.

## References

[1] Siegel R , Naishadham D , Jemal A . Cancer statistics for Hispanics/Latinos, 2012. CA Cancer J Clin. 2012;62:283. 22987332 10.3322/caac.21153

[2] Chen W , Zheng R , Baade PD , et al. Cancer statistics in China, 2015. CA Cancer J Clin. 2016;66:115. 26808342 10.3322/caac.21338

[3] Madersbacher S , Hochreiter W , Burkhard F , et al. Radical cystectomy for bladder cancer today–a homogeneous series without neoadjuvant therapy. J Clin Oncol. 2003;21:690‐696. 12586807 10.1200/JCO.2003.05.101

[4] Stenzl A , Cowan NC , De Santis M , et al. Treatment of muscle‐invasive and metastatic bladder cancer: update of the EAU guidelines. Actas Urologicas Espaolas. 2012;59:1009‐1018. 10.1016/j.acuro.2011.11.001 22386114

[5] Alexandroff AB , Jackson AM , O'Donnell MA , James K . BCG immunotherapy of bladder cancer: 20 years on. Lancet. 1999;353:1689‐1694. 10335805 10.1016/S0140-6736(98)07422-4

[6] Zeegers M , Tan F , Dorant E , van den Brandt PA . The impact of characteristics of cigarette smoking on urinary tract cancer risk: a meta‐analysis of epidemiologic studies. Cancer. 2000;89:630‐639. 10931463 10.1002/1097-0142(20000801)89:3<630::aid-cncr19>3.3.co;2-h

[7] Huang P , Chen J , Wang L , et al. Implications of transcriptional factor, OCT‐4, in human bladder malignancy and tumor recurrence. Med Oncol. 2012;29:829‐834. 21533858 10.1007/s12032-011-9962-4

[8] Galarneau A , Richard S . Target RNA motif and target mRNAs of the Quaking STAR protein. Nat Struct Mol Biol. 2005;12:691‐698. 16041388 10.1038/nsmb963

[9] Zhang R‐L , Yang J‐P , Peng L‐X , et al. RNA‐binding protein QKI‐5 inhibits the proliferation of clear cell renal cell carcinoma via post‐transcriptional stabilization of RASA1 mRNA. Cell Cycle. 2016;15:3094‐3104. 27767378 10.1080/15384101.2016.1235103 PMC5134695

[10] Ebersole TA , Chen QI , Justice MJ , Artzt K . The quaking gene product necessary in embryogenesis and myelination combines features of RNA binding and signal transduction proteins. Nat Genet. 1996;12:260. 8589716 10.1038/ng0396-260

[11] Yang G , Fu H , Zhang J , et al. RNA‐binding protein quaking, a critical regulator of colon epithelial differentiation and a suppressor of colon cancer. Gastroenterology. 2010;138(231–40):e1‐e5. 10.1053/j.gastro.2009.08.001 PMC2847771 19686745

[12] Biedermann B , Hotz HR , Ciosk R . The quaking family of RNA‐binding proteins: coordinators of the cell cycle and differentiation. Cell Cycle. 2010;9:1929‐1933. 20495365 10.4161/cc.9.10.11533

[13] Wang S , Yang Y , Xing W , Chen J , Liu C , Luo X . Altered neural circuits related to sustained attention and executive control in children with ADHD: an event‐related fMRI study. Clin Neurophysiol. 2013;124:2181‐2190. 23800705 10.1016/j.clinph.2013.05.008

[14] Bian Y , Wang LI , Lu H , et al. Downregulation of tumor suppressor QKI in gastric cancer and its implication in cancer prognosis. Biochem Biophys Res Commun. 2012;422:187‐193. 22569043 10.1016/j.bbrc.2012.04.138

[15] Fu X , Feng Y . QKI‐5 suppresses cyclin D1 expression and proliferation of oral squamous cell carcinoma cells via MAPK signalling pathway. Int J Oral Maxillofac Surg. 2015;44:562‐567. 25457822 10.1016/j.ijom.2014.10.001

[16] Lu W , Feng F , Xu J , et al. QKI impairs self‐renewal and tumorigenicity of oral cancer cells via repression of SOX2. Cancer Biol Ther. 2014;15:1174‐1184. 24918581 10.4161/cbt.29502 PMC4128860

[17] de Miguel FJ , Pajares MJ , Martínez‐Terroba E , et al. A large‐scale analysis of alternative splicing reveals a key role of QKI in lung cancer. Mol Oncol. 2016;10:1437‐1449. 27555542 10.1016/j.molonc.2016.08.001 PMC5423218

[18] Zhao YI , Zhang G , Wei M , et al. The tumor suppressing effects of QKI‐5 in prostate cancer: a novel diagnostic and prognostic protein. Cancer Biol Ther. 2014;15:108‐118. 24153116 10.4161/cbt.26722 PMC3938513

[19] Feber A , Clark J , Goodwin G , et al. Amplification and overexpression of E2F3 in human bladder cancer. Oncogene. 2004;23:1627‐1630. 14716298 10.1038/sj.onc.1207274

[20] Androulakakis PA , Davaris P , Karayannis A , et al. Urothelial tumors of the bladder. Child Nephrol Urol. 1992;12:32‐34. 1606579

[21] Huang LI , Luo J , Cai Q , et al. MicroRNA‐125b suppresses the development of bladder cancer by targeting E2F3. Int J Cancer. 2011;128:1758‐1769. 20549700 10.1002/ijc.25509

[22] Aggarwal BB , Sung B . NF‐kappaB in cancer: a matter of life and death. Cancer Discov. 2011;1:469‐471. 22586649 10.1158/2159-8290.CD-11-0260 PMC3392037

[23] Aggarwal BB , Vijayalekshmi RV , Sung B . Targeting inflammatory pathways for prevention and therapy of cancer: short‐term friend, long‐term foe. Clin Cancer Res. 2009;15:425‐430. 19147746 10.1158/1078-0432.CCR-08-0149

[24] Zhang HM , Sang XG , Wang YZ , Cui C , Zhang L , Ji WS . Role of Delta133p53 isoform in NF‐κB inhibitor PDTC‐mediated growth inhibition of MKN45 gastric cancer cells. World J Gastroenterol. 2017;23:2716‐2722. 28487608 10.3748/wjg.v23.i15.2716 PMC5403750

[25] Hellman LM , Fried MG . Electrophoretic mobility shift assay (EMSA) for detecting protein‐nucleic acid interactions. Nat Protoc. 2007;2:1849‐1861. 17703195 10.1038/nprot.2007.249 PMC2757439

[26] Oeggerli M , Tomovska S , Schraml P , et al. E2F3 amplification and overexpression is associated with invasive tumor growth and rapid tumor cell proliferation in urinary bladder cancer. Oncogene. 2004;23:5616‐5623. 15122326 10.1038/sj.onc.1207749

[27] Wu L , Timmers C , Maiti B , et al. The E2F1‐3 transcription factors are essential for cellular proliferation. Nature. 2001;414:457‐462. 11719808 10.1038/35106593

[28] Sethi G , Ahn KS , Aggarwal BB . Targeting nuclear factor‐kappa B activation pathway by thymoquinone: role in suppression of antiapoptotic gene products and enhancement of apoptosis. Mol Cancer Res. 2008;6:1059‐1070. 18567808 10.1158/1541-7786.MCR-07-2088

[29] Wang S , Liu Z , Wang L , Zhang, X . NF‐κB signaling pathway, inflammation and colorectal cancer. Cell Mol Immunol. 2009;6:327. 19887045 10.1038/cmi.2009.43 PMC4003215

[30] Huang H , Ma L , Li J , et al. NF‐κB1 inhibits c‐Myc protein degradation through suppression of FBW7 expression. Oncotarget. 2014;5:493‐505. 24457827 10.18632/oncotarget.1643 PMC3964224

[31] Liu JY , Dai YB , Li X , et al. Solute carrier family 12 member 5 promotes tumor invasion/metastasis of bladder urothelial carcinoma by enhancing NF‐κB/MMP‐7 signaling pathway. Cell Death Dis. 2017;8:e2691. 28333147 10.1038/cddis.2017.118 PMC5386524

[32] Zhu J , Li Y , Chen C , et al. NF‐κB p65 overexpression promotes bladder cancer cell migration via FBW7‐mediated degradation of RhoGDIα protein. Neoplasia. 2017;19:672‐683. 28772241 10.1016/j.neo.2017.06.002 PMC5540704

[33] Iwata N , Ishikawa T , Okazaki S , et al. Clinical significance of methylation and reduced expression of the quaking gene in colorectal cancer. Anticancer Res. 2017;37:489‐498. 28179294 10.21873/anticanres.11341

[34] Ramkissoon SH , Bandopadhayay P , Hwang J , et al. Clinical targeted exome‐based sequencing in combination with genome‐wide copy number profiling: precision medicine analysis of 203 pediatric brain tumors. Neuro Oncol. 2017;19:986. 28104717 10.1093/neuonc/now294 PMC5570190

[35] Shingu T , Ho AL , Yuan L , et al. Qki deficiency maintains stemness of glioma stem cells in suboptimal environment by downregulating endolysosomal degradation. Nat Genet. 2016;49:75. 27841882 10.1038/ng.3711 PMC5453714

[36] Darbelli L , Choquet K , Richard S , Kleinman CL . Transcriptome profiling of mouse brains with qkI‐deficient oligodendrocytes reveals major alternative splicing defects including self‐splicing. Sci Rep. 2017;7:7554. 28790308 10.1038/s41598-017-06211-1 PMC5548867

[37] Oeggerli M , Schraml P , Ruiz C , et al. E2F3 is the main target gene of the 6p22 amplicon with high specificity for human bladder cancer. Oncogene. 2006;25:6538‐6543. 16953223 10.1038/sj.onc.1209946

[38] Yu F , Jin L , Yang G , Ji L , Wang F , Lu Z . Post‐transcriptional repression of FOXO1 by QKI results in low levels of FOXO1 expression in breast cancer cells. Oncol Rep. 2014;31:1459‐1465. 24398626 10.3892/or.2013.2957

[39] Wang LI , Zhai D‐S , Ruan B‐J , et al. Quaking deficiency amplifies inflammation in experimental endotoxemiaviathe Aryl Hydrocarbon receptor/signal transducer and activator of transcription 1–NF‐κB pathway. Front Immunol. 2017;8:1754. 29276519 10.3389/fimmu.2017.01754 PMC5727050

[40] Chen C , Luo F , Yang Q , et al. NF‐κB‐regulated miR‐155, via repression of QKI, contributes to the acquisition of CSC‐like phenotype during the neoplastic transformation of hepatic cells induced by arsenite. Mol Carcinog. 2018;57:483‐493. 29240254 10.1002/mc.22772

