# Supplementary material for: QKI‐6 inhibits bladder cancer malignant behaviours through down‐regulating E2F3 and NF‐κB signalling
Source: J Cell Mol Med. 2019 Aug 26;23(10):6578–94. doi: 10.1111/jcmm.14481 (PMC6787450; doi:10.1111/jcmm.14481)
Supplement: Supplementary file 1 [file JCMM-23-6578-s001.doc]

**QKI-6 inhibits malignant behaviors through downregulating E2F3 and NF-κB signaling in bladder cancer**

**Supplementary Figure Legends**

**Figure S1. QKI-6 shRNA knockdown in bladder cancer cells.** (A) Western blot. Bladder cancer 5637 cells were grown and infected with lentivirus carrying two different QKI-6 shRNA constructs for 3 days and then subjected to Western blot analysis for QKI expression. (B-C) Immunofluorescence. Bladder cancer 5637 and T24 cells were grown and infected with lentivirus carrying QKI shRNA and cDNA, respectively for 3 days and then subjected to immunofluorescence assays（400×）.

**Figure S2. QKI-6 promotes tumor cell cycle arrest at G2/S phase and regulates expression of various protein expressions.** (A) Flow cytometric assay. Following QKI-6 knockdown in 5637 and QKI-6 overexpression in T24 cells, cells were grown and then subjected to flow cytometric cell cycle analysis. (B) Quantified data of A. The data are from three independent experiments and summarized as the mean ± SD. *P < 0.05 *vs*. the control. (C) Western blot. Following QKI-6 knockdown in 5637 and QKI-6 overexpression in T24 cells, cells were grown and then subjected to Western blot analysis of cyclinE1, cyclinD1, cyclinA1, cyclinB, p27, and p21. (D) Quantified data of C.

**Figure S3. Effects of QKI-6 overexpression and knockdown on regulation of QKI, Ki67, PCNA, and PARP expression in nude mouse tumor cell xenografts**. Nude mouse tumor cell xenografts were dissected and subjected to tissue processing and immunofluorescence detection of QKI (A), Ki67 (B), PCNA (C), and PARP (D) expression（200×）. (E) Quantified data of A - D.

**Figure S4. Effects of the NF-κB inhibitor PDTC on QKI-6-induced cell proliferation, apoptosis, cell cycle arrest, and invasion.** T24 cells transfected with vector and QKI-6 cDNA were subjected to apoptosis (A), cell cycle distribution (C), and proliferation (E) assays, while 5637 cells transfected with sh-NC and sh-QKI-6 plasmid were subjected to apoptosis (B), cell cycle distribution (D), and proliferation (F) assays. Each assay was repeated at least three times.

**Table S1.** Primers used for qPCR and siRNA sequences to knockdown gene expression

| **Gene name** | | **DNA sequences** |
| --- | --- | --- |
| **QKI-5** | | 5'-ATC CTA TTG AAC CTA GTG GTG TA-3' |
|  |  | 5'-GGT CAG AAG GTC ATA GGT TAG TT-3' |
|  | |  |
| **QKI-6** |  | 5'-ATC CTA TTG AAC CTA GTG GTG TA-3' |
|  |  | 5'-AGG GTT CAG TTA AGA CCG TTC T-3' |
|  | |  |
| **β-actin** | | 5'-TAC CAC TGG CAT CGT GAT GGA CTC-3' |
|  |  | 5'-GCT CGG TGA GGA TCT TCA TGA GGT-3' |
|  | |  |
| **E2F3** | | 5'-AGA AAG CGG TCA TCA GTA CCT-3' |
|  |  | 5'-TGG ACT TCG TAG TGC AGC TCT-3' |
|  | |  |
| **si-QKI-1** | | 5'-GGC ACC UAC AGA GAU GCC AAC AUU A-3' |
|  |  | 5'-UAA UGU UGG CAU CUC UGU AGG UGC C-3' |
|  | |  |
| **si-QKI-2** | | 5'-CCU UGA GUA UCC UAU UGA ACC UAG U-3' |
|  |  | 5'-ACU AGG UUC AAU AGG AUA CUC AAG G-3' |
|  | |  |
| **sh-QKI** | | 5'-CCT TGA GTA TCC TAT TGA ACC TAG T-3' |
| **QKI promoter chip** | | 5'-TAG TAG TTT CCA GCA CCG AAT T-3' |
|  |  | 5'-GCT GTC CAT CGC ATC ACA-3' |
| **QKI promoter** | | 5'-AGG GAA TAG TAA CTT GCA AAA AAG CTA TGG-3' |
|  |  | 5'-GGC GGC GCT GCC AGC GGC GGC GGC AGC AGC-3' |


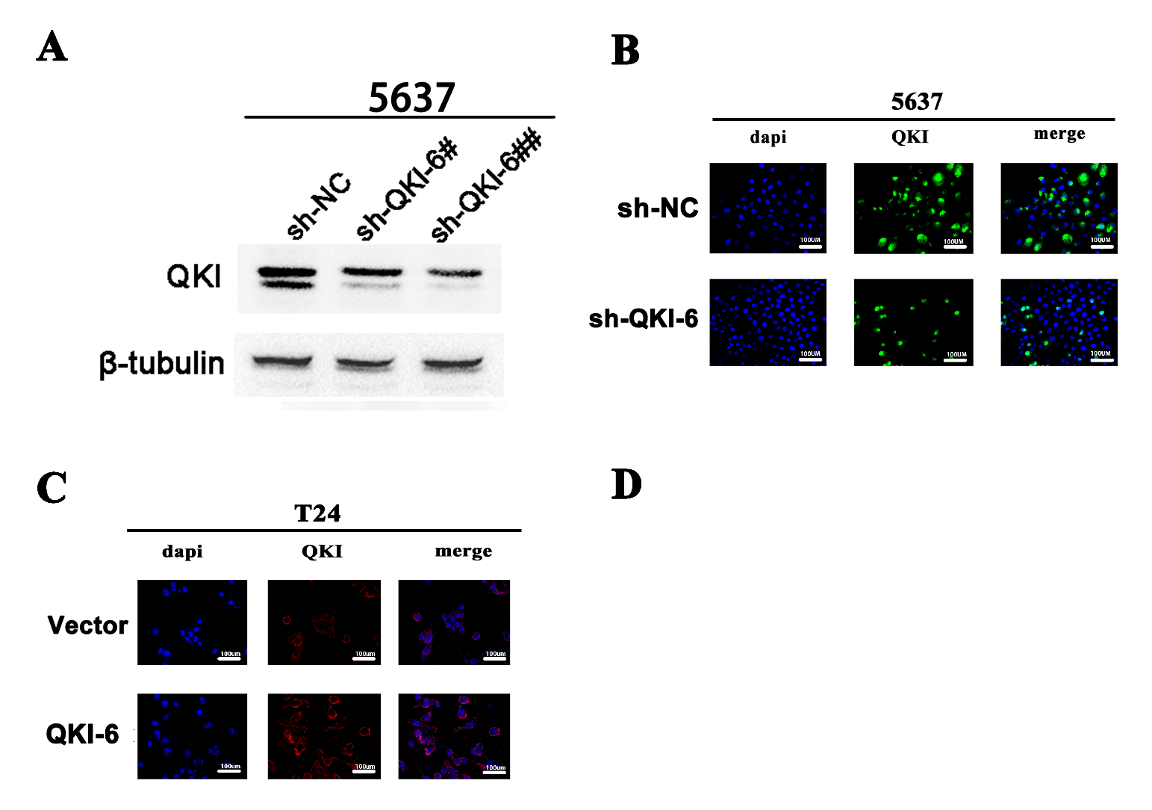


**Figure S1**


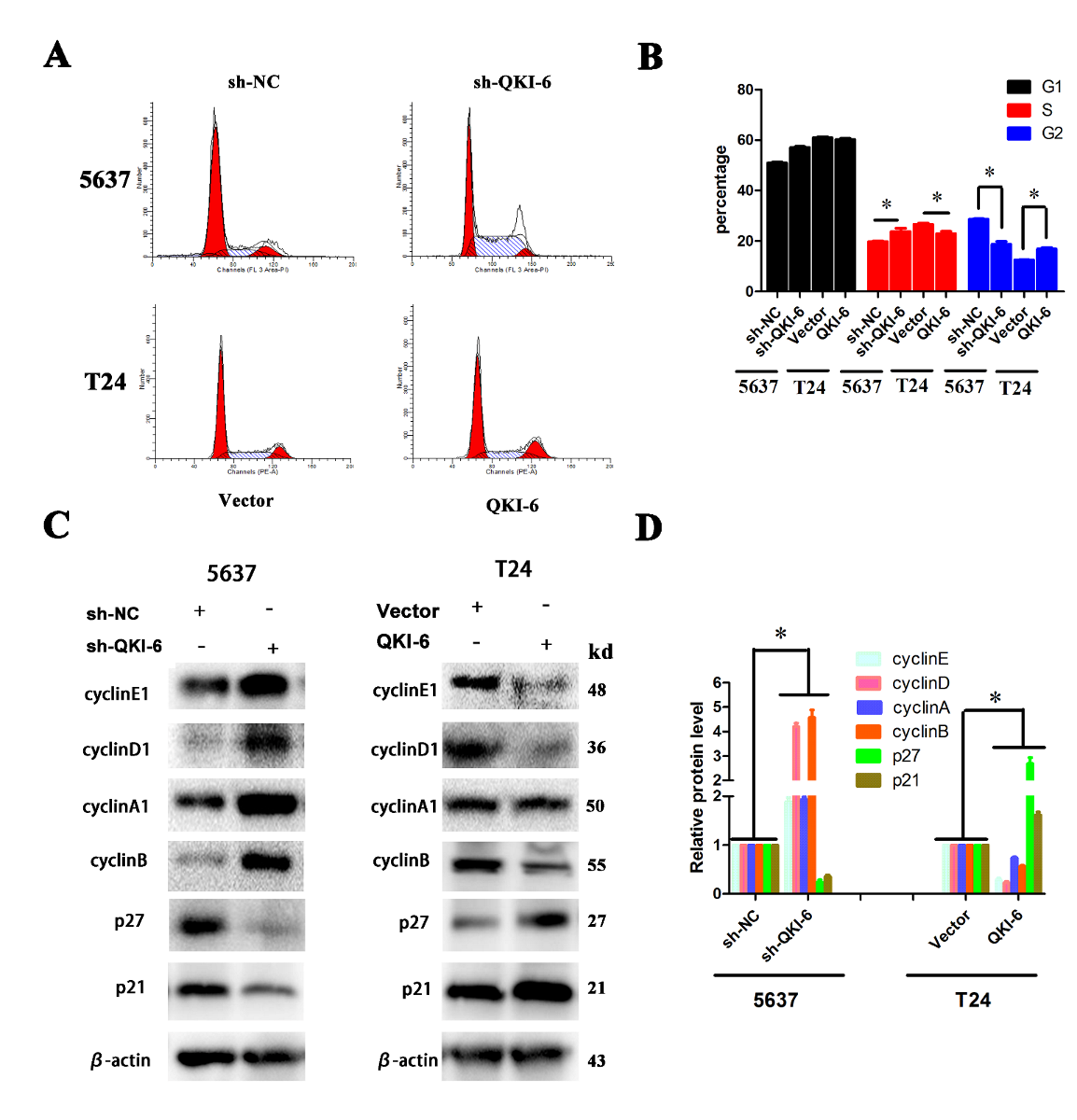


**Figure S2**


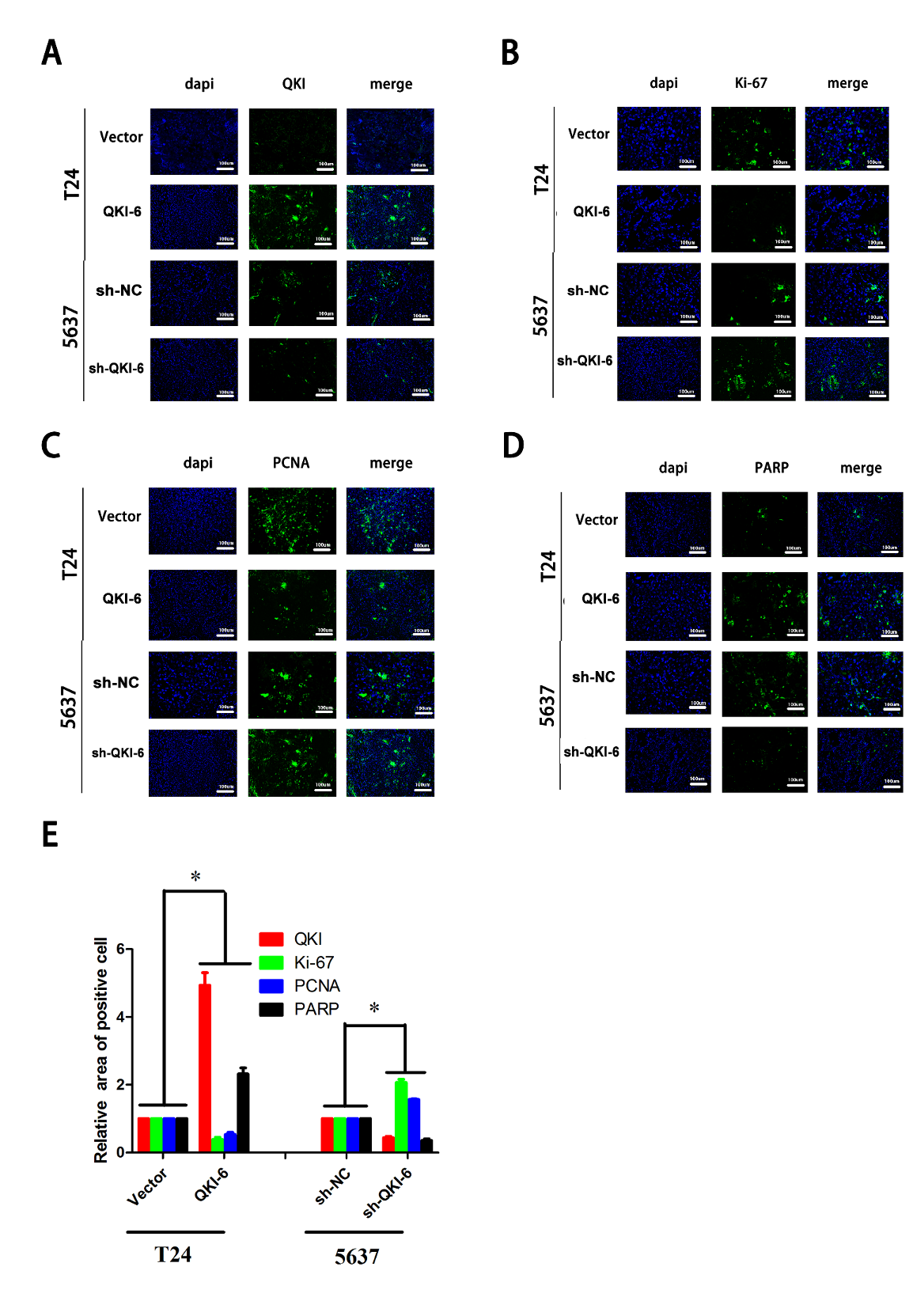


**Figure S3**


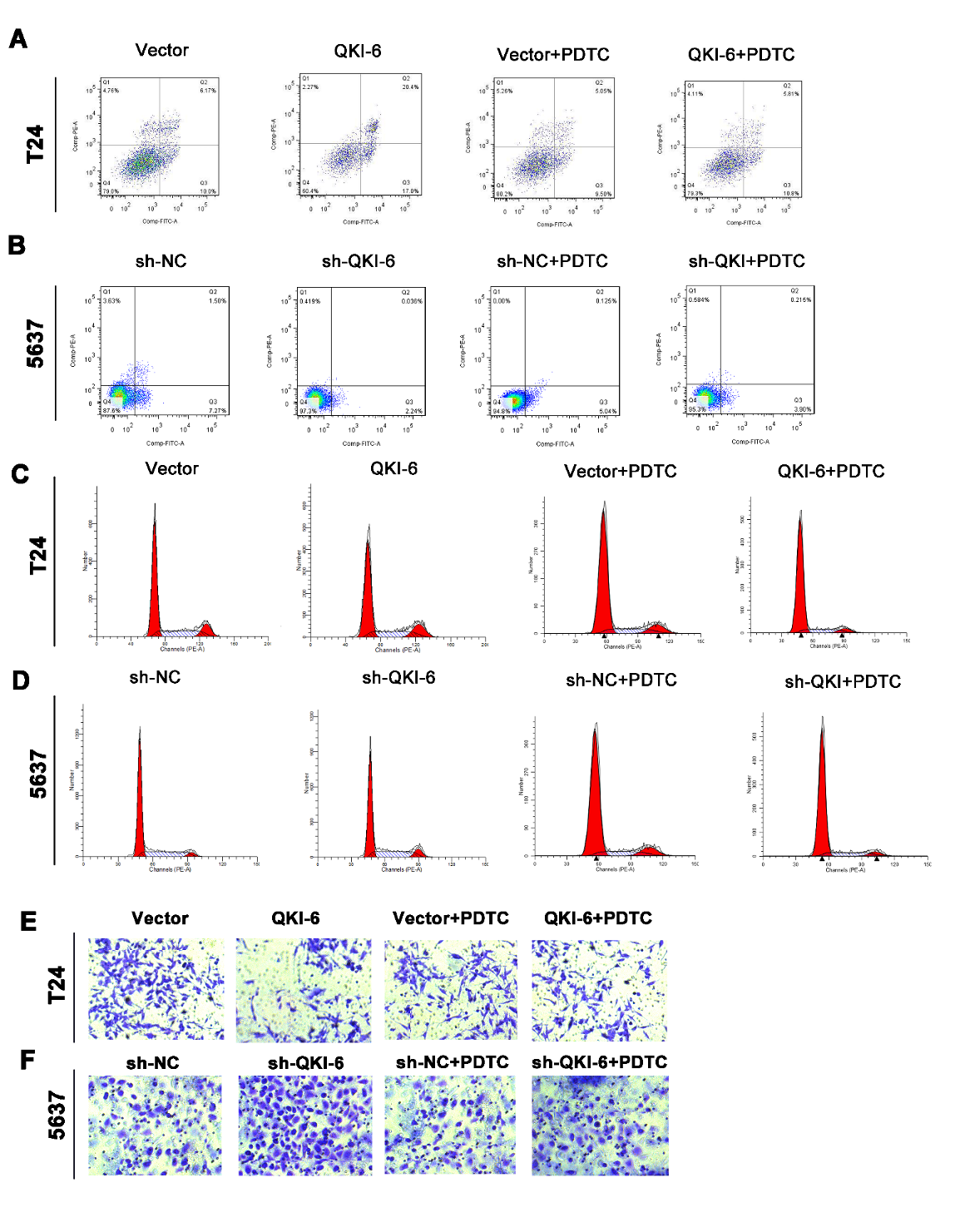


**Figure S4**
